# Identification of novel cancer therapeutic targets using a designed and pooled shRNA library screen

**DOI:** 10.1038/srep43023

**Published:** 2017-02-22

**Authors:** David Oliver, Hao Ji, Piaomu Liu, Alexander Gasparian, Ellen Gardiner, Samuel Lee, Adrian Zenteno, Lillian O. Perinskaya, Mengqian Chen, Phillip Buckhaults, Eugenia Broude, Michael D. Wyatt, Homayoun Valafar, Edsel Peña, Michael Shtutman

**Affiliations:** 1 University of South Carolina, College of Pharmacy, Department of Drug Discovery and Biomedical Sciences, Columbia, SC 29208, USA; 2 University of South Carolina, College of Arts and Sciences, Department of Statistics, Columbia, SC 29208, USA; 3 Bentley University, Waltham, MA 02452, USA; 4 University of South Carolina, College of Engineering and Computing, Department of Computer Sciences and Engineering, Columbia, SC 29208, USA

## Abstract

Targeted cancer therapeutics aim to exploit tumor-specific, genetic vulnerabilities specifically affecting neoplastic cells without similarly affecting normal cells. Here we performed sequencing-based screening of an shRNA library on a panel of cancer cells of different origins as well as normal cells. The shRNA library was designed to target a subset of genes previously identified using a whole genome screening approach. This focused shRNA library was infected into cells followed by analysis of enrichment and depletion of the shRNAs over the course of cell proliferation. We developed a bootstrap likelihood ratio test for the interpretation of the effects of multiple shRNAs over multiple cell line passages. Our analysis identified 44 genes whose depletion preferentially inhibited the growth of cancer cells. Among these genes ribosomal protein RPL35A, putative RNA helicase DDX24, and coatomer complex I (COPI) subunit ARCN1 most significantly inhibited growth of multiple cancer cell lines without affecting normal cell growth and survival. Further investigation revealed that the growth inhibition caused by DDX24 depletion is independent of p53 status underlining its value as a drug target. Overall, our study establishes a new approach for the analysis of proliferation-based shRNA selection strategies and identifies new targets for the development of cancer therapeutics.

Genetic heterogeneity of human cancers drives the need to develop a broad panel of therapeutics specifically targeting tumor cells. Therapeutic development depends on the exploration of genes and pathways critical for the growth and survival of cancer cells. The most important genes are those whose inhibition ultimately kills tumor cells while having minimal impact on normal tissues. The most direct approach for target gene identification is functional profiling with genome wide libraries of trans-dominant genetic inhibitors (TGIs). Several types of TGI libraries have been developed. These include genetic suppressor element (GSE) libraries^1^, which are libraries of short cDNA fragments expressing either anti-sense RNAs or inhibitory peptides^2^, small hairpin RNA (shRNA) libraries^3^ which are libraries expressing the stem and loop of shRNAs which are processed by cellular enzymes to double stranded inhibitory RNAs^4^, and more recently, single guide RNA (sgRNA) libraries^5^. The application of Next-Generation Sequencing (NGS) to the screening of TGI libraries allows quantitative analysis of screening results^3^,^6^. NGS-based screening procedures are designed to discover genes based on the analysis of enrichment or depletion of TGIs in cells subjected to selection, relative to non-selected cells. The major screening strategies for cancer therapeutic target identification are screens which detect the depletion of growth-suppressing TGIs over the course of cell propagation, or conversely, those which detect the enrichment of growth-suppressing TGIs^7^.

Most previously published functional screens are endpoint procedures^7^, where the abundance of TGIs is analyzed at the initial and final points only. However, NGS-based strategies allow the analysis of multiple data points to determine the kinetics of shifting TGI abundances. The latter approach, while it provides more information about biological processes, is often not used due to the difficulty of analyzing such complex data sets. Previously we performed BrdU suicide selection of a genome wide GSE library in a panel of normal and cancer cell lines to select tumor-specific target genes^8^. The BrdU suicide selection procedure enriches for growth-suppressing GSEs. That is, growth-suppressing GSEs provide survival advantage during this selection resulting in their over-representation at the end of the experiment. Through the initial analysis of this dataset we identified the *ζ*-subunit of the COPI complex (COPZ1)^8^. Inhibition of COPZ1 kills a majority of tumor cell lines, while not affecting the growth of normal cells. Here we re-sequenced and analyzed this dataset to reveal a large set of potential target genes. To reduce this list to the most effective targets, we designed an shRNA library focused on the identified gene set and performed a proliferation-based selection of the library in four tumor cell lines and normal fibroblasts. Enrichment or depletion of shRNAs were determined during the course of cell propagation. The screening results were then analyzed with a bootstrap likelihood ratio test (BLRT) statistical procedure we developed. This screen and analysis procedure revealed a subset of 44 genes with additional evidence for cancer-specific growth inhibition. The dependence of cancer cells on these genes was further tested with siRNAs against individual genes. Three genes successfully showed efficacy in all three selection procedures, ribosomal protein RPL35A, DEAD-box RNA helicase DDX24, and, in addition to the previously identified *ζ*-subunit, the *δ*-subunit of COPI, ARCN1. We show the association of the expression of the identified genes with development and mortality of human cancers and explore the mechanism of the effect of ARCN1 and DDX24 depletion in tumor cells.

## Results

### Genetic suppressor element library screen

Previously we constructed a library of GSEs composed of cDNA fragments from the human transcriptome (average length 135 bp) prepared from a mixture of normalized (reduced-redundancy) cDNAs from 18 cell lines derived from different types of cancers^8^. The library was subjected to BrdU-suicide selection (Supplemental Fig. 1) for identification of growth-suppressing GSEs in three tumor cell lines: MDA-MB-231 (breast cancer), PC3 (prostate cancer), and HT1080 (fibrosarcoma), as well as immortalized normal human foreskin fibroblasts, BJ-hTERT. The cDNA fragments were recovered and amplified from genomic DNA of BrdU selected and non-selected cells^8^. We performed additional sequencing of the library by 454 sequencing (Roche), obtaining approximately 100,000 GSE reads per sample. Target genes were identified by mapping fragments back to the human genome. 221 genes were targeted by at least 1 GSE enriched more than 1.5 fold in two or three tumor cell lines relative to normal cells (Supplemental Dataset S1). Gene Ontology based functional analysis showed that the set was enriched for genes involved in RNA splicing (18 genes) and translation (21 genes). Also, eight genes involved in the PI3K-AKT signaling pathway were identified using KEGG pathways analysis. The results of functional analysis are presented in Supplemental Tables S1,S2,S3,S4.

### Design, construction, and screening of a focused shRNA library to select growth inhibitory shRNAs

To analyze the cancer cell-specific efficacy of inhibiting the 221 genes identified in the GSE screen we designed a focused shRNA library. The main goal of the selection was to identify shRNAs depleted in proliferating tumor cell lines relative to normal fibroblasts. The shRNA library was designed to have six unique shRNAs against each of the 216 genes identified in the GSE data set. 6 unique shRNAs could not be designed for 5 of the identified genes (See Materials and Methods for details). Each shRNA was tagged with a unique barcode and an amplification adapter for rapid identification of unique shRNAs within the pool (Supplemental Fig. 2A; Supplemental Dataset S2). The shRNAs and barcodes were synthesized on-chip by parallel nucleotide synthesis, and subsequently inserted into the lentiviral vector.

**Figure 1:**
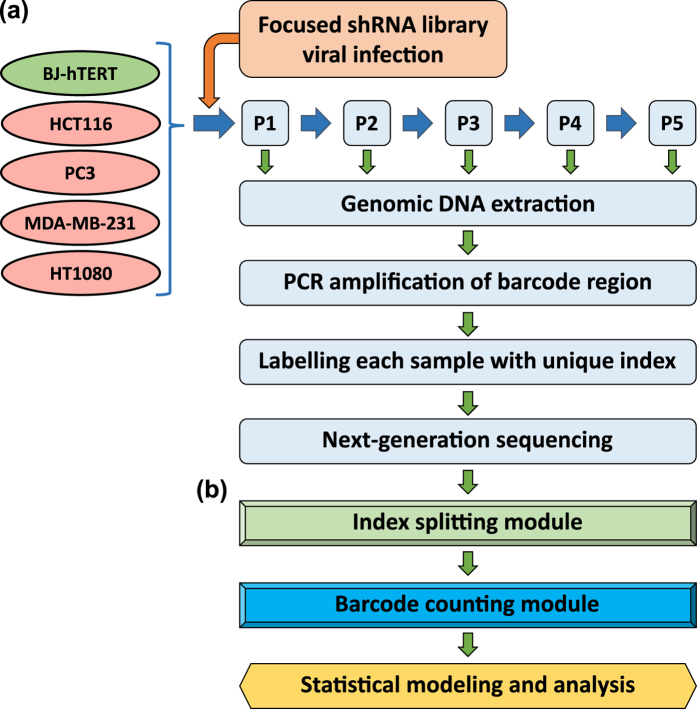
Experimental design of focused shRNA library screening and sequencing. (**a**) The focused shRNA library was infected into BJ-hTERT, PC3, MDA-MB-231, HCT116, and HT1080 cells. 48 hrs later cells were collected. One forth of the cells were re-plated (P1) and the rest were used for genomic DNA extraction. The procedure was repeated for 4 passages (P2-P5). During the 3-step barcode amplification (See Materials and Methods for details) unique index-sequences were added to amplified fragments from each sample. Resulting indexed libraries were mixed together and sequenced on an Illumina HiSeq 2500. (**b**) Sequencing data were processed first by identifying the sample index and then by identifying the specific shRNA barcode resulting in cell line, passage number, and shRNA specific reads. These data were then submitted to statistical analysis.

Normal fibroblast (BJ-hTERT), breast cancer (MDA-MB-231), colon cancer (HCT116), prostate cancer (PC3), and fibrosarcoma (HT1080) cell lines were infected with the library-containing lentiviruses in duplicates. The library-infected cells were passaged five times to undergo approximately 20 divisions (Fig. 1a). At each passage (approximately every fourth division) genomic DNA was isolated from a portion of the cells and the barcode-containing sequences were PCR amplified from the genomic DNA. During the second round of amplification, indexes were added to each library for multiplexed sequencing (Supplemental Fig. 2B). The resulting 50 libraries were pooled and sequenced at an average depth of 3 million reads per sample. Sequenced samples were split by index and unique shRNA barcodes were counted using a bespoke algorithm. Barcode read data were then used to analyze enrichment and depletion of shRNAs over passages (Fig. 1b). The amplification and index-splitting procedure we developed to allow parallel sequencing of as many pooled libraries as needed (far more than 24 pooled libraries allowed by standard procedure of Illumina).

### Development of a bootstrapped likelihood ratio test procedure to identify genes of interest

**Figure 2:**
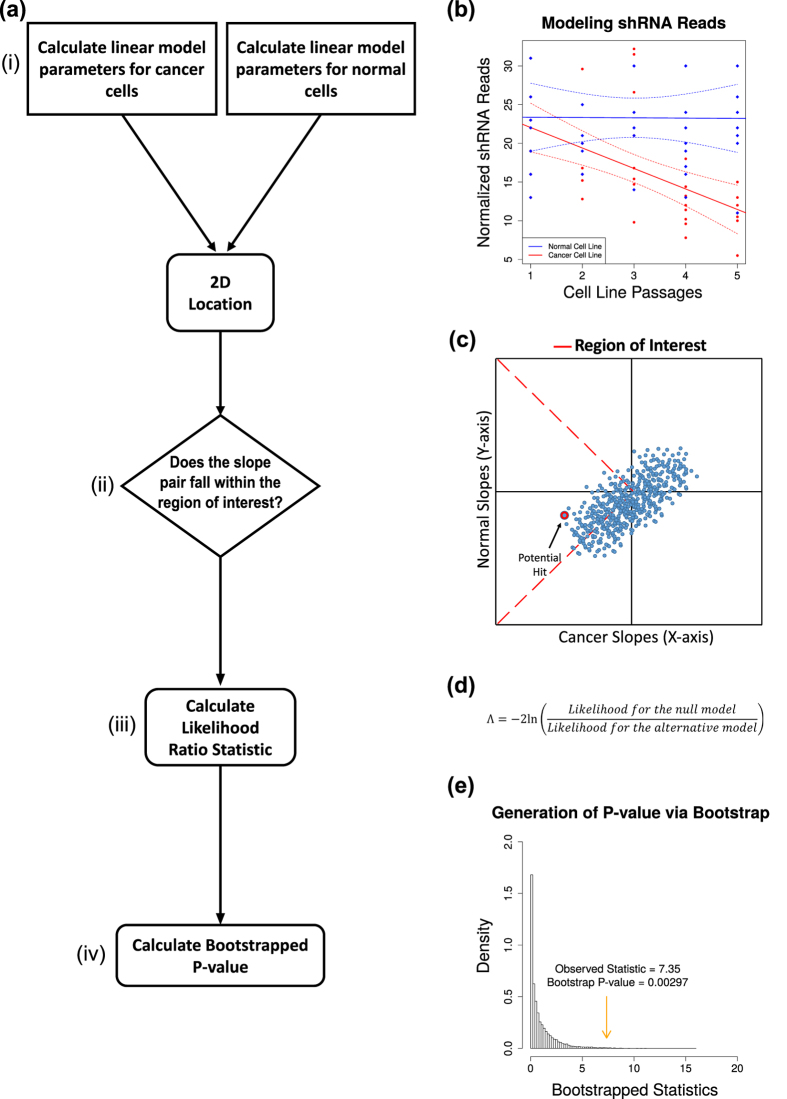
Statistical analysis of shRNA depletion over multiple passages. (**a**) The analysis of shRNA reads over multiple passages was performed by (i) calculating the linear regression model for the cancer cell line of interest and the normal cell line. (ii) shRNAs whose regression parameters fall within the region of interest were then identified. (iii) The likelihood ratio test statistic was calculated for shRNAs within the region of interest. (iv) A P-value for the likelihood ratio test statistic was then calculated via a bootstrap method. (**b**) A representation of the linear regression model calculated for all shRNAs targeting a single gene over five cell line passages for cancer (red) and normal (blue) cells. (**c**) The region of interest (demarcated in red) was defined by depletion of shRNA reads in cancer cell lines (x-axis) and less extreme depletion or enrichment in normal cells (y-axis). (**d**) The likelihood ratio test statistic for genes within the region of interest was the −2ln of the likelihood of the regression parameters being located outside the region of interest (null model) over the likelihood that the regression parameters were within the region of interest (alternative model). (**e**) A P-value for the observed likelihood ratio statistic, for a given target gene, was calculated as the probability of observing a value at least as large as the observed value out of 10,000 residual re-sampling bootstraps.

In order to analyze this dataset we developed a modified general linear model approach using normalized shRNA reads as the response variable and passages as a predictor (Fig. 2a(i)). This method for analysis allows us to identify shRNAs with cancer specific efficacy by comparing them to the control BJ-hTERT cell line without the need for specific sets of positive and negative control shRNAs.

We defined a region of interest where the slope of the fitted line for the six shRNAs against a gene in the cancer cell line being queried is less than zero while the corresponding slope for the shRNAs against the same gene in the normal cell line is not more extreme than the cancer cell line (Fig. 2a(ii)). This criterion allows for flexible normal cell line response to the shRNAs targeting the gene of interest while constraining the cancer cell line to depletion of the same shRNAs over passages. Specifically, shRNA barcode reads were normalized using upper-quantile normalization followed by Box-Cox transformation (*λ* ≈ 0.5). Since we used a general linear model, we checked that the normality assumptions were met before modeling (Supplemental Fig. 3). Once the model was fitted, we identified genes whose shRNAs affected cell growth such that the slope of the shRNA reads as a function of passage fell within the region of interest described above (Fig. 2a,b). If the estimated slopes were found to be within the region of interest, then we calculated the likelihood ratio test (LRT) statistic for that gene (Fig. 2a(iii)). To calculate a P-value for the gene-specific LRT statistic, we performed bootstrapping by re-sampling the residuals 10,000 times, calculating the LRT for each bootstrapped sample to determine the probability of observing a LRT statistic at least as extreme as the observed LRT statistic value (Fig. 2a(iv)). See methods section for detailed derivation of the BLRT.

### Identification of shRNAs that inhibit the growth of tumor cells

For the first three passages, the variability between the biological replicates was small and the correlations were strong. After passage 3, there was variability between the biological replicates that prevented any genes from passing our threshold for statistical significance (p < 0.05) after multiple testing corrections (Supplemental Fig. 4). The observed variability is likely caused by the cumulative impact of off-target effects of shRNAs^9^. However, 72 genes had some evidence for selective targeting (−2ln(LRT) >0) in the cancer cells compared to the normal control cell line through 5 passages. We stratified these genes by evidence in the four cancer cell lines. Genes in group 1 had a −2ln(LRT) >0 in all four cell lines. Genes in group 2 had a −2ln(LRT) >0 in 3 cell lines. Genes in group 3 had a −2ln(LRT) >0 in 2 cell lines. For group 4, since the evidence is only in a single cell line, the requirements were made more stringent, by requiring the LRT statistic to be greater than 0.1. Of the original 72 genes, 44 genes fell into at least one of these four categories (Supplemental Table S5). 26 of the 44 genes have previously been reported to be involved in cancer. Of the 6 genes in group 1, 4 were previously identified as having a role in cancer. Of the 9 genes in group 2, 5 have previously been identified as cancer related genes. 19 genes were identified in group 3, of which 12 have previously been reported to have an involvement in cancer. Finally, 10 genes were identified in group 4, 5 of which have previously been reported to be involved in cancer (Supplemental Table S6). The fact that many of these genes had been previously identified as having a role in cancer supports the validity of our method to identify potential cancer therapeutic targets.

### Secondary siRNA screens identify potential therapeutic targets

**Figure 3:**
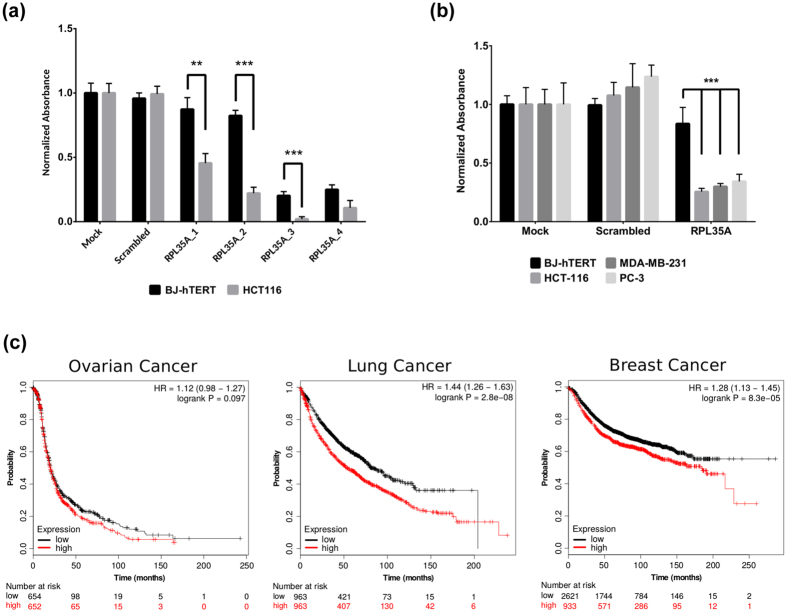
RPL35A depletion significantly reduces cancer cell growth compared to normal cells. (**a**) BJ-hTERT and HCT116 cells were transfected with four individual siRNAs, 5 nM each (Qiagen). The number of cells was quantified 5 days after transfection using SRB assay. Bars represent mean normalized absorbance at 540 nm +/− 1 SD (P-values: 0.0035, 7.6356e-05, 0.0009, N.S.). P-values were calculated using single tailed Welch’s t-test. (**b**) BJ-hTERT, HCT116, MDA-MB-231, and PC-3 cell lines were transfected with 2.5 nM of pooled siRNAs (GE Healthcare). Cell number determined and represented as in (**a**) (P-values: all <0.0001). Significance calculated as above but with additional Holm-Sidak correction for multiple testing. All siRNA transfections presented on panel A and B performed in 4 replicates. (**c**) Association of RPL35A expression in Affimetrix array datasets with survival of ovarian, breast, and lung cancer patients was analyzed using KM-plotter^70^. Plots represent survival of patients with high (red) and low (black) level of RPL35A expression.

**Figure 4:**
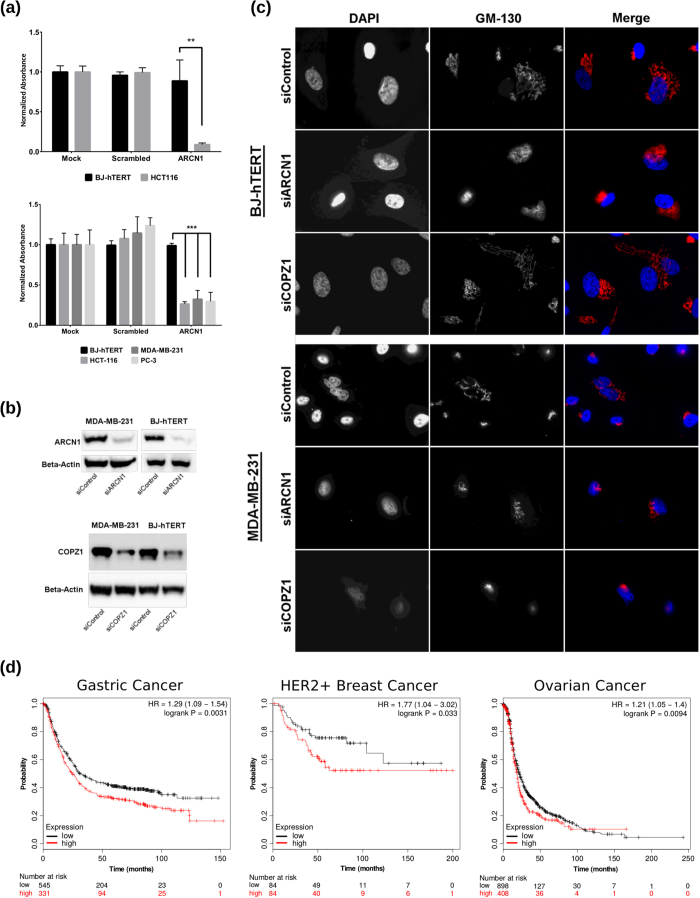
ARCN1 is a potential target for cancer therapy. (**a**) BJ-hTERT and HCT-116 were transfected with 5 nM individual siRNA (Qiagen) (Upper Panel). BJ-hTERT, MDA-MB-231, HCT-116, and PC3 cells were transfected with 2.5 nM of pooled siRNAs (GE Healthcare) (Lower Panel). Cell number, representation, and statistical tests were done as in Fig. 3. P-values are <0.0063 and All <0.0001 for Upper and Lower Panels respectively. All siRNA transfections performed in 4 replicates. (**b**) ARCN1 and COPZ1 expression were analyzed by WB in MDA-MB-231 and BJ-hTERT cells. Cells were transfected with control, ARCN1, or COPZ1 siRNAs (2.5 nM, GE Healthcare pooled siRNAs). Cells were lysed 72 hrs post-transfection followed by WB analysis of *β*-Actin, ARCN1, or COPZ1 proteins with corresponding antibodies. (**c**) BJ-hTERT and MDA-MB-231 cells were transfected with COPZ1, ARCN1, and control siRNA (as in (**b**)). Golgi and cell nuclei were visualized by IF with anti-GM130 and DAPI. Depletion of ARCN1 results in Golgi disruption (no distinct tubular structure) in both normal (BJ-hTERT) and cancer cells (MDA-MB-231), while depletion of COPZ1 by siRNA results in selective disruption of the Golgi in cancer cells without affecting the Golgi in normal cells. (**d**) Association of ARCN1 expression with survival of gastric, breast, and ovarian cancer patients. Kaplan–Meier analysis performed as described in Fig. 3.

**Figure 5:**
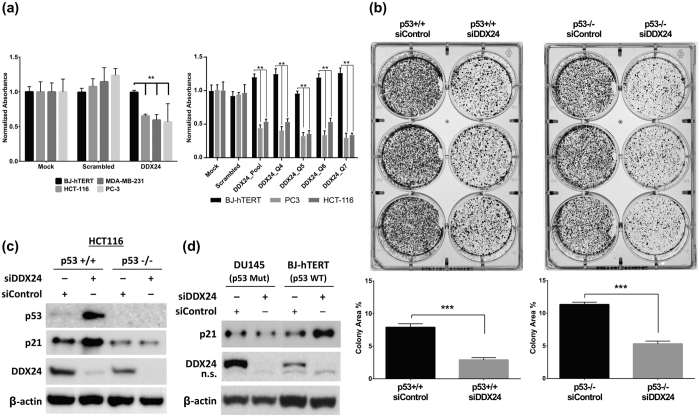
DDX24 mediated inhibition of cancer cell growth is independent of p53. ((**a**) Left panel) BJ-hTERT, HCT116, MDA-MB-231, and PC-3 cell lines were transfected with 2.5 nM pooled DDX24 siRNAs (GE Healthcare) in quadruplicates. Cell number, representation, and statistical tests as in Fig. 3 (P-values: 0.0026, 0.0004, and 0.0002, respectively). ((**a**) Right panel) BJ-hTERT, HCT116, and PC3 cells were transfected with 5 nM of pooled DDX24 siRNA (GE Helthcare) and 4 individual siRNAs (Qiagen) in 6 replicates. Cell number, representation, and statistical tests as in Fig. 3 (P-values < 0.0001). (**b**) HCT-116 with p53 wild-type (p53+/+) and p53 null (p53−/−) were transfected with 5 nM of control or DDX24 siRNAs and plated for colony formation assay. Cells were transfected in triplicate and plated at 1,000 cells per well followed by quantification of total colony area. Bars represent average +/− 1 SD (P-values: 0.0005 and <0.0001, respectively). (**c**) Western blot analysis of p53, p21, and DDX24 expression in wild-type and p53−/− HCT-116 cells. Cells were transfected with 5 nM of siRNAs and lysed 72 hrs post-transfection followed by WB with corresponding antibodies. (**d**) Western blot analysis of p21 and DDX24 expression in DU145 and BJ-hTERT cells. Cells were transfected with 5 nM of pooled siRNAs and lysed 72 hrs post-transfection followed by WB with corresponding antibodies.

The genes identified in the focused shRNA screen were subjected to further analysis to reveal whether short-term depletion of the gene would provide a similarly discriminatory effect between tumor and normal cells as that observed with the longer shRNA treatment. To this end, the effects of siRNA knockdown of the 44 selected genes were assessed in HCT-116, MDA-MB-231, PC-3, and BJ-hTERT cells. The cells were transfected with pooled siRNAs (GE Healthcare) in 96 well format, followed by quantification of the cell number five days after transfection (Supplemental Table S9). The siRNA depletion of 3 genes - ARCN1, DDX24, and RPL35A - significantly inhibited the growth of all three cancer cell lines but not normal cells. The growth inhibitory effects of pooled siRNAs were confirmed by the depletion of the genes with different individual siRNAs from another source (Qiagen) (Figs 3a,b, 4a and 5a). ARCN1 was identified as a group 1 gene by the analysis of shRNAs screening, and DDX24 and RPL35A were identified as group 3 genes (Supplemental Table S7).

### Depletion of RPL35A preferentially inhibits growth of tumor cells and its expression correlates with poor survival of cancer patients

RPL35A is a part of the large ribosomal subunit. The depletion of RPL35A with both pooled and four individual siRNAs preferentially inhibited growth of tumor cell lines (Fig. 3a,b). The cytotoxic effects observed in cancer and normal cells for 2 of the individual siRNAs (Qiagen) could be attributed to off-target effects. Analysis of gene expression profiles deposited in GEO shows that high expression level of RPL35A strongly correlated with poor survival of breast, ovarian, and lung cancer patients (Fig. 3c). Furthermore, in the majority of cancer datasets available in TCGA, the expression level of RPL35A mRNA is higher than in corresponding normal tissues (Supplemental Fig. 7).

### Effects of ARCN1 depletion on growth and Golgi integrity

The ARCN1 gene encodes the *δ*-COP protein which is an integral component of the COPI complex^10^,^11^. Previously we showed that depletion of another subunit of the COPI complex, *ζ*1-COP protein, caused Golgi disruption followed by cell death in the majority of tumor cell lines but not in normal cells^8^. To compare the mechanisms of selective growth inhibition of tumor cells we analyzed the results of *δ*-COP depletion in MDA-MB-231 breast carcinoma cells and BJ-hTERT fibroblasts (Fig. 4b). Contrary to the results obtained from *ζ*1-COP depletion, *δ*-COP siRNAs induced Golgi disruption in both MDA-MB-231 and immortalized fibroblasts (Fig. 4c, Supplemental Figs 5 and 6), while affecting the growth of cancer cells only (Fig. 4a). The disruption of Golgi is characterized by complete or partial disappearance of tubular structures identified by staining with Golgi-specific marker GM130 (Supplemental Fig. 6). Furthermore, the analysis of ARCN1 expression in human tumors shows that higher levels of ARCN1 expression correlates with lower survival rate of gastric, ovarian, and HER+ positive breast cancer patients (Fig. 4d). Interestingly, in the majority of cancer datasets available in TCGA the expression level of ARCN1 mRNA are not significantly changed relative to corresponding normal tissues (Supplemental Fig. 7) suggesting that the sensitivity of cancer cells to ARCN1 depletion may not be dependent on increased ARCN1 expression.

### Depletion of DDX24 inhibits growth of tumor cells regardless of p53 status

DDX24 is a member of the DEAD-box family of putative RNA helicases^12^ which have been implicated in diverse cellular functions^13^,^14^,^15^,^16^,^17^,^18^. However, to our knowledge, this is the first identification of DDX24 as a target for selective inhibition of tumor growth (Fig. 5a). Our initial results showed that depletion of DDX24 with pooled siRNAs (GE Helthcare) inhibits the growth of tumor cells with differing p53 status, including HCT116 with a normal level of the wild-type p53, MDA-MB-231 with mutated p53, and PC3 which are p53 null^19^. These results were confirmed with the set of four individual siRNAs (Qiagen) (Fig. 5a). Furthermore, siRNA-mediated DDX24 depletion in parental (p53+/+) and p53−/− HCT116 cells also inhibits the growth of both wild type and p53−/− cells with similar efficacy (Fig. 5b), while elevating p53 and p21 expression in p53+/+ cells and not p53−/− cells (Fig. 5c). Similar results were observed with DU145 cells expressing p53-223Leu and p53-274Phe mutant proteins^19^ (Fig. 5d). Finally, DDX24 knockdown induced p21 expression in BJ-hTERT normal fibroblasts (Fig. 5d) without having significant effect on cell growth (Fig. 5a).

**Figure 6:**
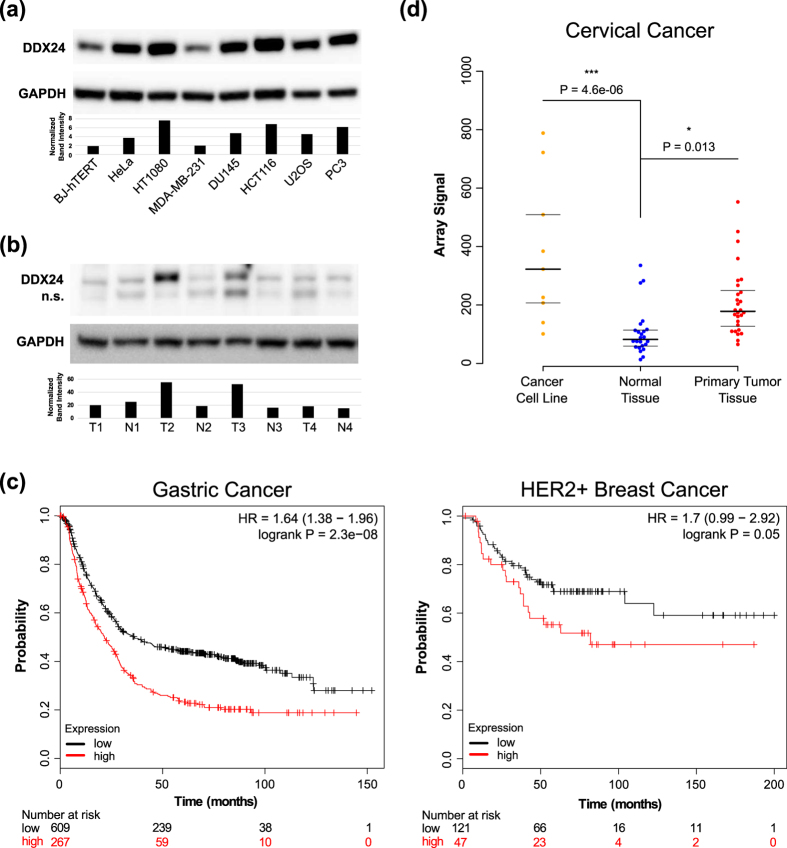
DDX24 expression is upregulated in cancer and correlates with survival. (**a**) Western blot analysis of DDX24 expression in BJ-hTERT, HeLa, HT1080, MDA-MB-231, DU145, HCT116, U2OS, and PC3 cell lines. Bars represent normalized DDX24/GAPDH intensity. (**b**) Western blot analysis of DDX24 expression in 4 pairs of matched tumor and normal colon tissue. Bars represent normalized DDX24/GAPDH intensity. (**c**) Association of DDX24 expression with survival of gastric and HER2 positive breast cancer patients. The Kaplan–Meier analysis was performed as described in Fig. 3. (**d**) The expression level of DDX24 in cervical cancer cell lines, primary tumors, and benign tissues^75^ (microarray data deposited in GEO database). P-values, calculated using single tailed Welch’s t-test with Holm-Sidak correction for multiple testing, are indicated for significant differences between the groups.

The level of DDX24 protein is elevated in all but one of the tumor cell lines we tested relative to BJ-hTERT fibroblasts (Fig. 6a). Moreover, DDX24 protein expression is significantly upregulated in two out of four samples of colon cancer tissues relative to normal counterparts (Fig. 6b). Analysis of expression of DDX24 in human tumors suggests that high level of the gene expression correlates with a lower survival rate of Gastric and HER2+ positive breast cancer patients (Fig. 6c). Furthermore, the level of DDX24 expression is elevated in cervical cancer tissues and cell lines, relative to normal tissues (Fig. 6d). In the majority of cancer datasets available in TCGA the expression level of DDX24 mRNA is not significantly changed relative to corresponding normal tissues (Supplemental Fig. 7). The conflicting RNA-seq (TCGA) and Western blotting results for DDX24 suggests a post-transcriptional regulation of DDX24 expression in cancer cell lines and tumor tissues.

## Discussion

We have developed a screening and analytical discovery protocol to identify novel genes required for growth and survival of tumor cells but not normal cells. The procedure employs (i) screening of a full genome GSE library, (ii) validation of the selection results with a designed shRNA library, and (iii) additional validation with gene-specific siRNAs. All screens were performed on a panel of tumor cell lines of different origin (prostate, breast, colon, and connective tissues) and normal fibroblasts. The negative selection pressure against elements of the focused shRNA library was ascertained through NGS-based monitoring of shRNA-specific reads over the course of multiple passages. To statistically analyze the effects of several shRNAs (6 per gene) over 5 passages per cell line in biological replicates, we developed a procedure utilizing a linear regression approach. During the development of this paper, an alternative approach that adapts the edgeR software^20^ for differential gene expression analysis to perform this type of analysis was reported^21^.

Re-sequencing and analysis of previously performed GSE library selection^8^ identified 221 genes targeted by growth inhibitory GSEs. Genes were selected for follow-up if their GSEs were enriched more than 1.5 fold in two or more tumor cell lines but not in normal fibroblasts. Follow-up validation with a focused shRNA library confirmed 44 genes; 26 of these had been previously linked to cancer development and progression (Supplemental Table S6). The selective dependency of tumor cells on the expression of the genes was further tested with individual siRNAs. The experimental procedure for siRNA-based analysis consisted of a short, 5-day treatment which was strikingly different than the shRNA library selection (5 passages over 16 to 25 days). It is therefore not surprising that depletion of only three genes – ribosomal protein RPL35A, COPI subunit ARCN1, and RNA helicase DDX24 – selectively inhibited the growth of all three cancer cell lines without strong effects on BJ-hTERT cells. The shorter duration of siRNA depletion provides additional strength to the importance of these validated targets.

The RPL35A gene encodes a 110 aa protein that functions as a part of the large ribosomal subunit, which is composed of 47 proteins. The RPL35A protein is not a part of the ribosome core structural proteins conserved along all taxons^22^; however, it is found in archaea (RPL35Ae) and all eukaryotes from vertebrates to yeast (RPL33 in Saccharomyces Cerevisiae)^23^. The yeast RPL35A homologue is involved in rRNA processing, 60S subunit assembly, and 60S to 40S subunit joining in the final step of translational initiation^24^. The depletion of RPL33 in yeast drastically inhibited protein biosynthesis and growth, while the phenotype could be partially reversed by overexpression of tRNA-Met^24^. The depletion of RPL35A in zebrafish embryos by egg-injection with morpholino antisense oligos resulted in abnormal phenotypes during embryo development^25^. Haploinsufficiency and mutations of RPL35A are associated with Diamond-Blackfan anemia, an autosomal dominant bone marrow failure syndrome^26^,^27^,^28^. The example of Diamond-Blackfan anemia, where mutations of different ribosomal proteins specifically affect the growth of highly proliferative erythroid progenitor cells, is a natural model of selective sensitivity of highly proliferating tumor cells to RPL35A depletion. Elevated RPL35A expression was found in glioma cells relative to normal astrocytes^29^, while RPL35A overexpression in Jurkat cells (an acute T-cell leukemia derived cell line) produced a cell death resistant phenotype^30^. The resistance of Jurkat cells overexpressing RPL35A was not accompanied by changes in the expression levels of Bcl-2 or Bcl-xL, suggesting a more complex mechanism for resistance than simple alterations in Bcl-dependent apoptotic signaling. Moreover, RPL35A was shown to cooperate with c-Myc in the transformation of pre-B cells^31^. shRNA-mediated depletion of RPL35A inhibits growth and promotes apoptosis of leukemic cell lines^26^, which is similar to our results. The molecular mechanism of differential sensitivity of tumor and normal cells is yet to be identified; however, the restrictive effect of RPL35A depletion in zebrafish embryo as well as haploinsufficiency in humans described above suggests the existence of a protective mechanism. In general, tumor cells are more sensitive to impairment of ribosomal biogenesis and associated stress, which has been suggested as an exploitable dependency for the development of new cancer treatments^32^. These results combined with our identification of RPL35A as a potent growth inhibitor in three independent cancer cell lines and the correlation of the poor survival of ovarian, breast, and lung cancer patients with the high expression of RPL35A, suggest that RPL35A is a key factor for survival of cancer cells, while normal cells are better protected from the effects of RPL35A depletion.

ARCN1 encodes the *δ*-subunit of the COPI complex. The COPI complex is composed of seven subunits with two of them, *ζ* and *γ*, encoded by two highly homologous isoforms each. The complex is one of three major vesicles coating protein complexes, Clathrin, COPII, and COPI. COPI-coated vesicles shuttle cargo within the Golgi organelle and from the Golgi back to the ER^33^ and are involved in autophagosome formation^34^. The disruption of COPI by inhibition of any of the unique subunits results in Golgi collapse and block of autophagy^35^,^36^. Interestingly, the deficiency of different COPI subunits translates to different phenotypes. Impairment of COPA leads to hereditary autoimmune-mediated lung disease and arthritis^37^, while ARCN1 mutations cause craniofacial abnormalities^38^. The mechanism of selective effects of inhibition of different subunits of the COPI complex needs to be identified. Previously we showed the mechanism of selective sensitivity of tumor cells to the inhibition of the *ζ*-subunit of the COPI complex, encoded by two homologous genes, COPZ1 and COPZ2. The depletion of COPZ1 specifically kills tumor cells, in which COPZ2 is downregulated, while normal cells are much less sensitive to COPZ1 depletion due to of the expression of COPZ2, which maintains COPI complex function in the absence of the *ζ*1-isoform^8^,^35^. Contrary to the depletion of COPZ1, where COPZ2 expression protects normal cells from Golgi disruption, depletion of ARCN1 disrupted the Golgi equally in normal and tumor cells. Therefore, the observed differential sensitivity of the normal fibroblasts and tumor cell lines to ARCN1 depletion, as well as the previously described sensitivity of non-small cell lung cancer cells bearing KRAS mutations^39^, is likely mediated by a different mechanism. It could possibly be attributed to differential dependence on COPI-dependent lipolysis^40^,^41^; however, extensive additional studies will be needed to identify the precise molecular mechanism of selective inhibition of tumor cell growth by ARCN1 depletion. The importance of ARCN1 for tumor progression is further confirmed by the association of high ARCN1 expression with the poor survival of gastric cancer patients and patients with HER2-positive breast cancer. Overall the results with ARCN1 confirmed our previous finding that targeting the COPI complex is a viable strategy for new cancer therapy.

DDX24 is the most promising target we identified due to potential druggability of the DDX24 protein. DDX24 belongs to a diverse family of DEAD-box RNA helicases (DDXs) that are present in all eukaryotic cells, as well as many bacteria and archaea. According to UniProt there are at least 80 members of the DDX family in human cells. When examined biochemically, family members possess coordinated ATPase and helicase activities and are extensively involved in RNA metabolism^42^. Specifically, DEAD-box RNA helicases appear to be involved in transcription, translation, ribosomal biogenesis, RNA degradation, and small RNA processing^42^,^43^. Recently, a small molecule inhibitor of DDX3 was developed and shown to attenuate HIV production^44^. In parallel, accumulating evidence is establishing the role of DDX3 and the other members of the family (DDX1, DDX2, and DDX58) as positive regulators of cancer development and progression^45^,^46^. The small molecule inhibitor of DDX3 targets the ATPase and helicase activity of DDX3 and was reported to inhibit cell growth and promote tumor regression in a murine model of lung cancer^47^. DDX24 is involved in multiple biological processes including HIV-1 infection^13^,^48^, interferon signaling^15^,^16^, and rRNA processing^14^,^49^. Importantly, DDX24 interacts with MDM2, p300, and p53^14^,^17^,^18^,^50^,^51^. Our results along with another recent report^18^ show that depletion of DDX24 activates p21 expression in a p53 dependent manner. However, we report here that siRNA depletion of DDX24 equally inhibited the growth of both p53 positive and p53 negative tumor cell lines. The mechanisms of growth inhibition by DDX24 depletion have yet to be identified and may involve defects in ribosomal RNA biogenesis^14^,^49^. In line with our results, inhibition of ribosomal biogenesis induces elevation of the p53 protein^52^. Analysis of publicly available expression profiles of human tumors showed that high level of DDX24 expression correlates with decreased survival of HER2 positive breast cancer and gastric cancer patients. Moreover, significant elevation of the level of DDX24 protein was detected in the majority of tumor cell lines of different origins and in 50% of colon cancer tissues we tested. These results are further confirmed by recent results from another group that show the elevation of DDX24 expression in many breast cancer cell lines^18^. Taken together these results suggest that DDX24 is a promising target for the development of therapy for a variety of tumors.

Collectively, our results show that the approach based on analysis of shRNA depletion in tumor cell lines relative to normal fibroblasts over multiple data points allows for the identification of genes necessary for cancer cell survival, and the identified genes are promising targets for further development of cancer therapeutics.

## Materials and Methods

### Cell lines and human tissues

MDA-MB-231, HCT116, HT1080, HeLa, U2OS, DU145, 293FT, and PC3 cell lines were obtained from American Type Culture Collection. HCT116 p53−/− (clone 379.2)^53^ line was a gift of Dr. B. Vogelstein (Johns Hopkins University). BJ normal foreskin fibroblasts, immortalized with human telomerase reverse transcriptase (line BJ1-hTERT) were obtained from Clontech Laboratories. HeLa, U2OS, 293FT, DU145, MDA-MB-231, HCT116, HCT116 p53−/−, HT1080, and PC3 cell lines were grown in DMEM with 10% FC-2. BJ fibroblasts were maintained in BJ medium (4:1 DMEM/M199) supplemented with 1 mmol/L of sodium pyruvate and 10% FBS. Flash frozen tumor and adjacent normal colon tissue samples were obtained from the Center for Colon Cancer Research Tissue Biorepository at the University of South Carolina (USC) with oversight and approval from the USC institutional review board. All samples were consented for use in biomedical research at the time of surgery. All the tissue samples and associated data obtained from the biorepository are fully deidentified, therefore materials are considered to be exempt from Human Subject regulations. As such, the presented experiments are not required to have IRB approval.

### Genetic suppressor element screen

Construction of the genome-wide GSE library and BrdU suicide selection were described previously^8^. To identify genes which were positively selected in this procedure, CLC workbench was used to map GSEs to the reference genome (hg19 downloaded from http://hgdownload.soe.ucsc.edu/goldenPath/hg19/bigZips/). Peaks were called using the CLC workbench ChIP-seq module (https://www.qiagenbioinformatics.com/). The closest gene to a significant (P-value < 0.05) peak was identified (if the peak was not located within a gene then the gene to which the peak was closest was chosen). The total number of reads in each peak were then normalized to the library size and enrichment was calculated by the ratio of cancer cell reads to normal cell reads. Genes with an enrichment greater than 1.5 in 2 or more tumor cell lines and/or targeted with 2 or more GSEs were chosen for further analysis.

### Design and construction of focused shRNA library

The focused shRNA library was intended to contain 6 shRNAs against each target gene such that: (i) each shRNA was 19 bp long, (ii) each duplex stability was between −32 and −28 ΔG, and (iii) each shRNA uniquely targeted the gene of interest (no other gene had greater homology) using published algorithm^54^. The resulting library contained 1,273 shRNAs, including 6 shRNA per gene for 209 genes, 5 shRNAs for one gene, 4 shRNAs for one gene, 3 shRNAs for two genes, 2 shRNAs for two genes, 1 shRNA for one gene (Supplemental Dataset S2). Each shRNA was assigned an error-correcting barcode sequence, which withstands sequencing errors designed as described in ref. 55. See Supplemental Fig. 2A for the scheme of the oligonucleotide design and Supplemental Dataset S2 for the complete sequences of the oligonucleotides. The shRNA and barcode-containing oligonucleotides were synthesized on-chip (Mycroarray, Ann Arbor MI), 25,000 oligonucleotides per chip followed by droplet PCR amplification with oligo-specific primers. The oligonucleotide mixture was digested with Bpi I (NEB, Ipswich, MA) and inserted into pRSI9-U6-(sh)-UbiC-TagRFP-2A-Puro lentiviral vector (Cellecta, Mountain View, CA). The library DNA was amplified as described previously^8^.

### Lentiviral infections

Lentiviral transduction was carried out as described^56^ using pCMV-Δ8.9 and pVSV-G packaging constructs. The vector plasmids pCMV-Δ8.9, pVSV-G, and DNA were mixed at 5:4:1 ratio and cotransfected into 293 FT cells using the polyethylenimine protocol^57^. Lentivirus-containing supernatants were harvested three times, at 24, 48, and 72 hours after transfection. Lentiviral library infection was carried out as previously described^6^.

### Selection of the shRNA library

Recipient cell lines were infected with the shRNA library in duplicates with a multiplicity of infection (MOI) of 0.9. Infected cells were cultivated over the course of five passages. On each passage one forth of the cells were re-plated and the rest of the cells were used for purification of genomic DNA. Genomic DNA was purified as previously described^6^. Barcode sequences were amplified from the DNA with three rounds of PCR (Supplemental Fig. 2B), 10 cycles each with the following primer pairs: FwdHTS/RevHTS, FwdGex/Rev-Ind (1:50 ratio), and FwGex/Seq2N-AD (See Supplemental Table S7 for primer sequences and Supplemental Table S8 for index sequences). The amplified products were purified with a PCR purification kit (Qiagen, Valencia, CA). Unique 8 bp indexes assigned to each library were incorporated in the second round of PCR and Illumina sequencing adapters were incorporated in the third round of PCR amplification (Supplemental Fig. 2B).

### Sequencing

50 bp, single end (SE) sequencing was performed at Cornell Epigenomics Core Facility using Illumina HiSeq2000. Resulting reads consisted of shRNA specific barcodes in FASTQ format. FASTQ data were processed in R^58^ using an in-house algorithm. Briefly, reads were split by index to produce FASTQ files containing sequences specific to a cell line, passage, and biological replicate. Reads were then assigned to a specific shRNA by identifying the barcode sequence within the read allowing for only a single mismatch.

### siRNA validation

Validation of hits from GSE and shRNA screens was performed using four different siRNAs from Qiagen (Human genome siRNA Library v1.0) and 4 pooled siRNAs from GE Healthcare (ON-TARGET plus SMARTpool). Four DDX24 specific siRNAs (not included into v1.0 above), SI04189073, SI04138064, SI04132135, SI00361214 were obtained from Qiagen. siRNA transfection was performed using a reverse transfection method. Briefly, siLentFect (Bio-Rad, Hercules, CA) was premixed with Opti-MEM media (ThermoFisher Scientific, Waltham, MA) at 0.075 *μ*l siLentFect per 20 *μ*l of Opti-MEM according to manufacturer protocol. siRNAs were added such that the final concentration was 5 nM (Qiagen) or 2.5 nM (GE Healthcare) and the siLentFect/Opti-MEM/siRNA mixture was incubated at room temperature for 20–30 min. Cells were added to premixed siRNA/siLentFect complexes at a seeding density of 5,000 cells per well in 96 well plate. siRNA treated cells were grown for 5 days at 37 °C and 5% CO_2_.

### Cell number quantification

Cell numbers were analyzed using the sulforhodamine B (SRB) assay as described^59^. Briefly, plated cells were fixed with 10% TCA at 4 °C, followed by washing with water. Fixed cells were stained with 0.4% SRB solution followed by washing with 1% acetic acid and drying of the SRB stained cells. SRB was solubilized in 20 mM Tris (pH 10) and quantified by measuring absorbance of SRB solution at 540 nm minus absorbance at 630 nm (background absorbance).

### Colony formation assay

siRNA-transfected cells were plated at 2,500 cells per well in 6 well plates followed by cultivation for 10 days. Colonies were fixed and stained with crystal violet fixing solution (0.25% Crystal Violet, 3.7% formaldehyde, 10% H_2_O, 80% methanol) as described^60^. Stained plates were imaged with the ChemiDoc Touch Imaging System (Bio-Rad) and resulting images were quantified using the Colony Area Plugin^61^ for ImageJ^62^.

### Western blot

To prepare total protein extracts for Western blot analysis, cells (3 × 10^5^ − 10^6^ per sample) were lysed according to standard procedures in TNT lysis buffer (20 mM Tris HCl, pH 7.5, 200 mM NaCl, 5 mM EDTA, 1% Triton X-100), supplemented with 1 mM DTT and Pierce Protease Inhibitor cocktail (Thermo Scientific, Cat. No. 88266). Total protein extracts from flash frozen tissue (5 mg per sample) were crushed followed by homogenization in TNT buffer. Protein concentration was measured using the Pierce BCA protein assay kit (Thermo Scientific, Cat. No. 23227) according to the manufacturer’s protocol. Protein expression (20 *μ*g per sample) was analyzed by Western Blotting with primary antibodies: p53 (DO-1, BD Biosciences, Cat. No. 554293), p21 (H-164, Santa Cruz Biotechnology, Cat. No. sc-756), DDX24 (Bethyl Laboratories, Cat. No. A300-698A-T), ARCN1 (GeneTex, Cat. No. 103252) GAPDH (Santa Cruz Biotechnology, sc-32233), and *β*-Actin (Thermo Scientific, Cat. No. MA5-15739) followed by corresponding (HRP)-conjugated secondary antibodies (Thermo Scientific, Cat. Nos 31460, 31430). Membranes were cut into strips containing different molecular weight areas to allow simultaneous analysis of multiple proteins. Each membrane fragment was probed separately and stripped and re-probed if necessary. The digital images were obtained using the ChemiDoc Touch Imaging System (Bio-Rad, Hercules, CA). Complete copies of the WBs are presented in Supplemental Fig. 8.

### Fluorescent microscopy

Cells cultured on glass coverslips (Bellco Glass) were fixed with fresh 3.7% paraformaldehyde and permeabilized with 0.5% Triton X-100. Fixed and permeabilized cells were blocked with 3% (BSA) and incubated with mouse anti-GM130 primary antibody (BD Biosciences, 610822) and DAPI (Invitrogen). The secondary antibody was Cy3-conjugated goat anti-mouse IgG (Jackson, 711-165-152). Fluorescence images were acquired with a PlanApo/N 60/1.42 NA objective on an Olympus IX81 microscope. Hamamatsu C10600 camera gain and exposure time settings were controlled with Metamorph Basic. Processing of images (merging, brightness, and final size) was performed using Fiji software^62^,^63^.

### Gene ontology

Gene ontology analysis was performed using the Gene Ontology Consortium website’s enrichment analysis tool^64^,^65^ or Kegg Mapper Pathways tool^66^.

### Association of gene expression with survival of cancer patients

Association of expression of the genes in GEO deposited Affymetrix data sets with relapse-free survival of breast cancer patients, progression free survival of ovarian cancer patients, and overall survival of gastric and lung cancer patients was determined using KM-plotter online survival analysis tool (http://kmplot.com/analysis/) with “Auto select best cutoff” option^67^,^68^,^69^,^70^.

### RNA-seq data of the gene expression in tumor and normal tissues

The expression of RPL35A, DDX24, and ARCN1 mRNAs was obtained from publicly available data from TCGA Firehose^71^, which represents RNA expression data from 38 cancer types and 19,125 samples.

### Statistical analysis of siRNA results

For siRNA toxicity, we used 4 biological replicates and performed two sided Welch’s t-tests with Holm-Sidak correction for multiple testing when necessary. T-tests and multiple testing corrections were performed with GraphPad Prism (GraphPad Software, La Jolla CA).

### Bootstrap LRT to test regression coefficients lying in a cone (shRNA screening analysis)

shRNAs of interest were measured in both normal and cancerous cell lines at different passages. Assuming shRNA measurements are independent and identically distributed normal random variables, a simple linear regression (SLR) is performed to estimate the rate of change in the mean shRNA reads over multiple passages for each type of cell populations. Let 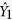 and 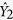 denote the estimated mean normalized shRNA reads for the cancer and normal cell lines, respectively. Let X_1_ and X_2_ denote the covariate vectors that indicate passage number for the measurements in the experiment. Let *n*_1_ and *n*_2_ denote the number of unique shRNAs targeting a gene in cancer and normal cell lines respectively. For 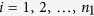, and 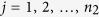, the fitted values of the shRNA reads are


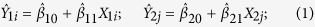



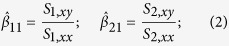



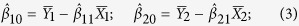



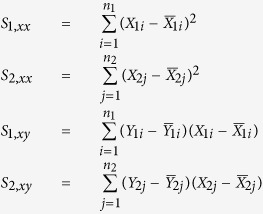


The goal of the study is to test whether the (*β*_11_, *β*_21_)-pair lies within the region of interest, which is the region of 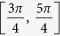 on a 2D plane formed by using *β*_11_ as the x-axis, and *β*_21_ as the y-axis. A bootstrap likelihood ratio test (BLRT) is constructed to compare slopes of the two regression lines in equation (1). We assume equal variances in both normal and cancerous shRNA measurements, denoted by *σ*^2^. *σ*^2^, *β*_10_ and *β*_20_ are then considered as nuisance parameters in this BLRT and we can form the following hypothesis:


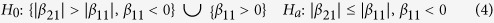


That is, under the null hypothesis the absolute value of the slope of the regression for the normal cell line (|*β*_21_|) is more extreme than the absolute value of the slope of the regression for the cancer cell line (|*β*_11_|) when the slope of the cancer cell line is less than 0 or the slope of the line for cancer cells is greater than 0 (indicating that the shRNA is more effective at reducing the survival/proliferation of normal cells than cancer cells or is ineffective at reducing the survival/proliferation of cancer cells).

Under the alternative hypothesis the absolute value of the slope of the regression for the normal cell line is less extreme than the absolute value of the slope of the regression for the cancer cell line when the cancer cell line slope is less than 0.

Let Θ_*r*_ denote the restricted parameter space,


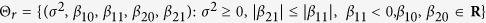


Let Θ denote the unrestricted parameter space,


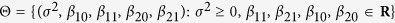


The likelihood ratio *λ*(**Y**_1_, **Y**_2_) is then the likelihood of the null model over the likelihood of the unrestricted model,


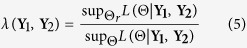


where the likelihood of the data is


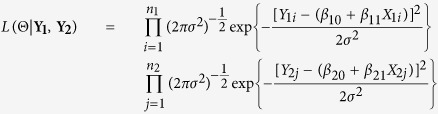


In equations (1) and (2), we obtain unrestricted parameter estimators for the regression equations. The restricted estimators are obtained by maximizing the likelihood of the data over the restricted parameter space Θ_*r*_. The estimators are shown below

1. When the unrestricted Maximum Likelihood Estimates (MLEs) for *β*_11_ and *β*_21_ fall in the 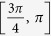 region, then the restricted MLEs of *β*_11_ and *β*_21_ satisfy the property that 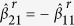, that is, they reside on the upper boundary of the region of Θ_*r*_. As such the restricted estimates of all the parameters are given by
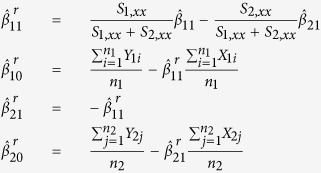
2. When the unrestricted MLEs for *β*_11_ and *β*_21_ fall in the 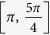 region, then the restricted MLEs of *β*_11_ and *β*_21_ satisfy the property that 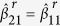, that is, they reside on the lower boundary of the region of Θ_*r*_. As such the restricted estimates of all the parameters are given by

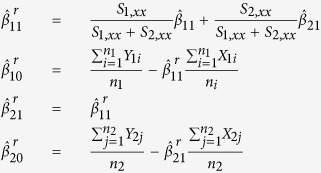


We then derive the negative log of the LRT statistic using equation (5)


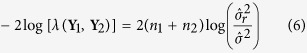


where 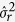 is the estimated variance under the null and 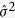 is the estimated unrestricted variance, defined as


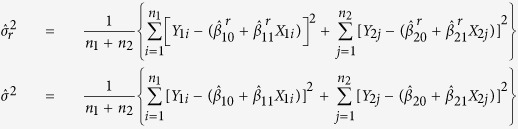


Denote the value of the test statistic from a observed data sample *T*_0_. To obtain a P-value for the LRT, *T*_0_, we approximate the null sampling distribution of the likelihood ratio test statistic via bootstrapping. We do this via the following procedure:

1. Obtain SLR residuals *e*_1*i*_, 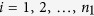 and *e*_2*j*_, 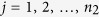 for both the normal and cancerous cell lines in the sample. 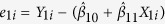; 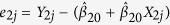, where 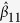, 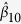, 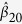, 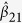 are the unrestricted maximum likelihood estimates (MLE) of the regression parameters.
2. Combine vector 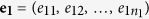 and 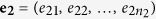. So, the combined vector is **e** = (**e**_**1**_, **e**_**2**_).
3. Obtain two samples of bootstrap residuals of size *n*_1_ and *n*_2_ from **e**. Call the two samples of residuals **S**_1_ and **S**_2_. **S**_1_ and **S**_2_ each is a random sample from **e**.
4. Using restricted estimates, create a bootstrap sample 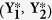 by adding non-restricted residuals in step 1. For example, for 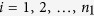, 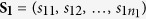,
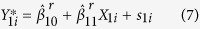
5. Compute the test statistic as in equation (6), say 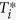 for sample 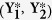 using the proposed negative log of the LRT.
6. Repeat steps 1–5, and compute a large number, say M = 1000, such test statistics.
7. The computed bootstrap p-value is given by 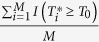. This p-value is used to test the *H*_0_ vs the *H*_1_.

This analysis was performed in R^58^, using the MASS^72^, reshape2^73^, and preprocessCore^74^ packages. The code for the statistical analysis presented here is available for download at https://github.com/doliv071/Focused_shRNA_Analysis.git.

## Additional Information

**How to cite this article**: Oliver, D. *et al*. Identification of novel cancer therapeutic targets using a designed and pooled shRNA library screen. *Sci. Rep.* **7**, 43023; doi: 10.1038/srep43023 (2017).

**Publisher's note:** Springer Nature remains neutral with regard to jurisdictional claims in published maps and institutional affiliations.

## Supplementary Material

Supplemental Information

Supplementary Dataset 1

Supplementary Dataset 2
