# Supplementary material for: Identification of novel cancer therapeutic targets using a designed and pooled shRNA library screen
Source: Sci Rep. 2017 Feb 22;7:43023. doi: 10.1038/srep43023 (PMC5320502; doi:10.1038/srep43023)
Supplement: Supplemental Information [file srep43023-s1.pdf]

Supplemental Information

Identification of novel cancer therapeutic targets using a designed and pooled shRNA library screen

David Oliver[1,+], Hao Ji[1,+], Piaomu Liu[2,4], Alexander Gasparian[1], Ellen Gardiner[1], Samuel Lee[1], Adrian Zenteno[1], Lillian O Perinskaya[1], Mengqian Chen[1], Phillip Buckhaults[1], Eugenia Broude[1], Michael D Wyatt[1], Homayoun Valafar[3], Edsel Pe˜na[2], and Michael Shtutman[1,*]

[1]University of South Carolina, College of Pharmacy, Department of Drug Discovery and Biomedical Sciences, Columbia, SC 29208, USA
[2]University of South Carolina, College of Arts and Sciences, Department of Statistics, Columbia, SC 29208, USA
[3]University of South Carolina, College of Engineering and Computing, Department of Computer Sciences and Engineering, Columbia, SC 29208, USA
[4]Bentley University, Waltham, MA 02452, USA
[*]shtutmanm@sccp.sc.edu
[+]these authors contributed equally to this work

Supplemental Figures 1 – 8
Supplemental Tables S1 – S9

Supplemetal DataSets S1,S2

Supplementary Figure 1: Genetic Suppressor Element selection procedure

(a)

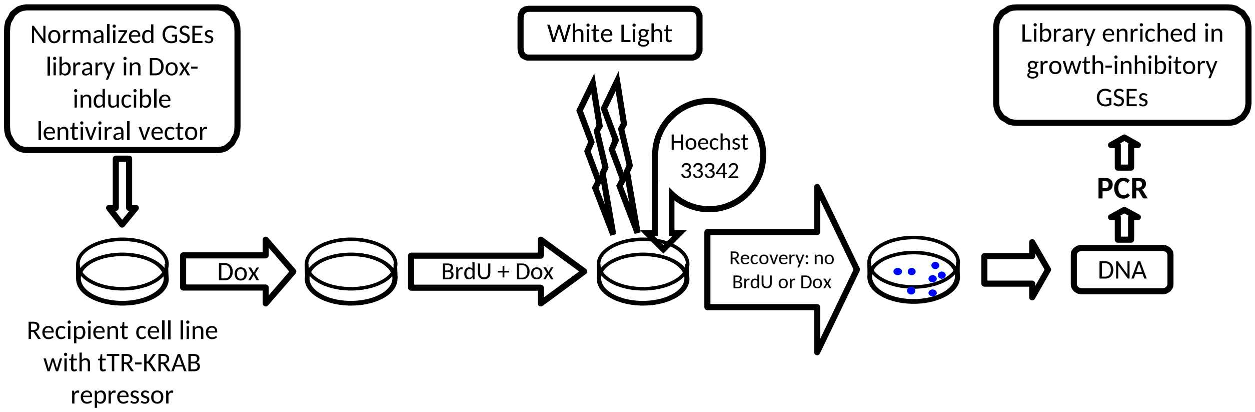

The selection enriches for arrested cells, while eliminating growing cells

(b)

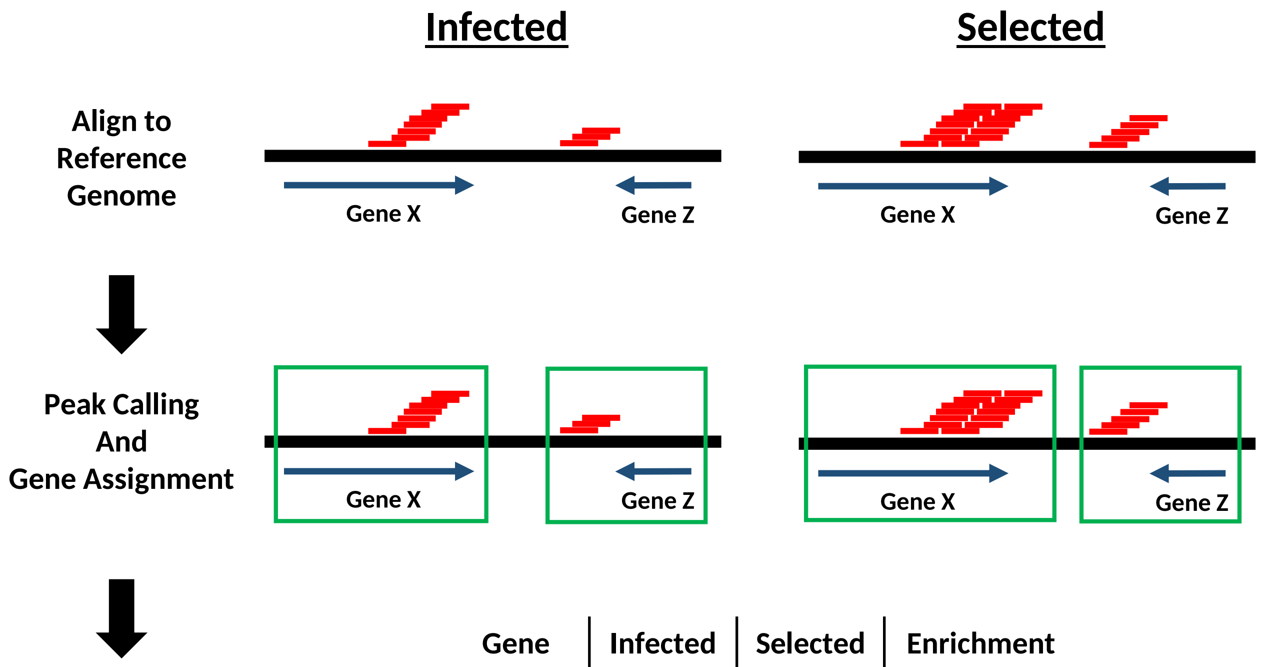

Calculate Enrichment

Gene	Infected	Selected	Enrichment
Gene U	59	30	0.51
Gene V	69	26	0.38
Gene W	55	42	0.76
Gene X	33	75	2.27
Gene Z	25	40	1.60

Selected for shRNA Screen

(a) Genetic suppressor element-containing, Doxicyclin-inducible vectors are infected into a panel of normal and cancer cell lines. GSE-vector expression is induced by Doxicyclin (DOX) treatment and then submitted to BrdU suicide selection for 72 hrs. BrdU-containing DNA is cross-linked with the addition of Hoechst 33342 and white light. DOX and BrdU are then removed to allow cells to recover. Finally, GSEs are recovered from genomic DNA through PCR amplification and sequencing. (B) Recovered GSEs from infected (no selection) and selected cells are aligned to the reference genome and peaks are called with CLC Workbench (Qiagen). Peaks are assigned to the nearest gene and reads per gene are quantified. Genes with > 1.5 fold enrichment in selected over infected were used for focused shRNA screen.

Supplementary Figure 2: shRNA library design and recovery

(a)

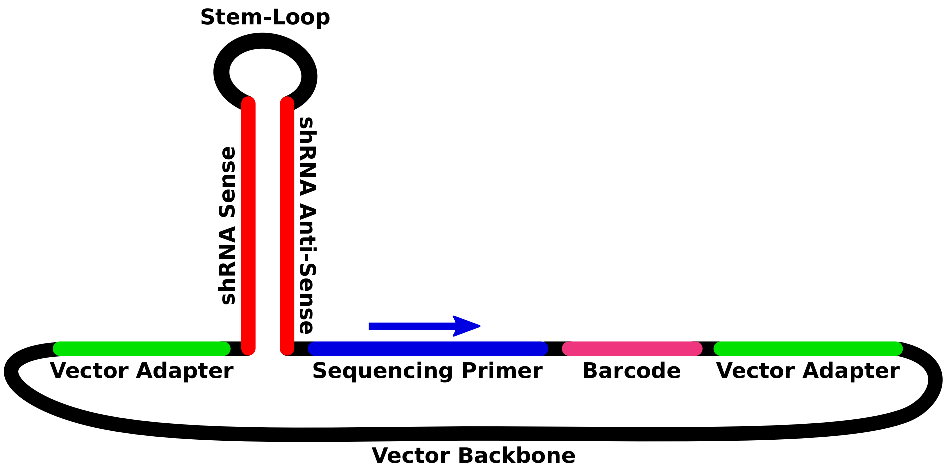

(b)

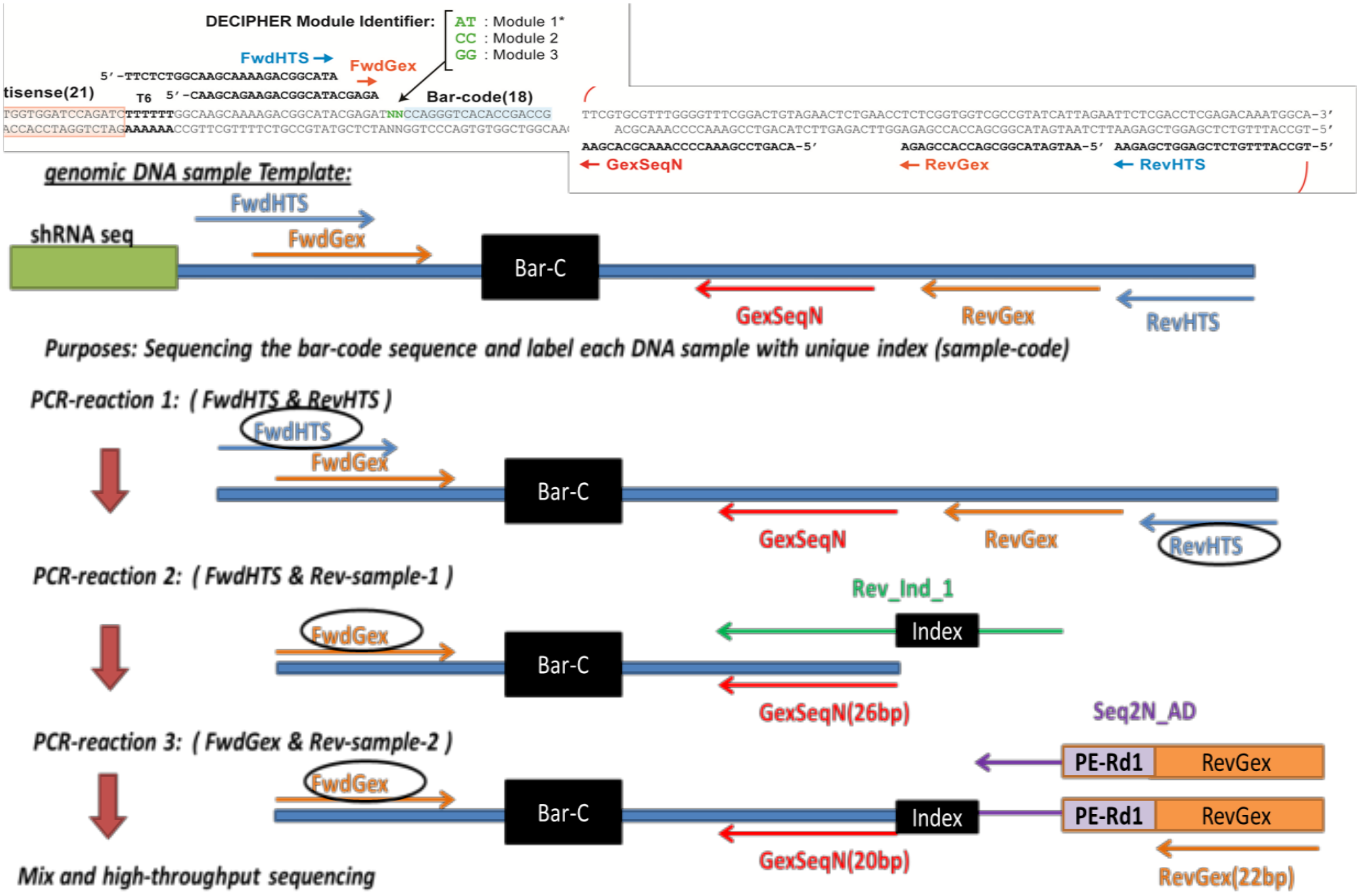

(a) Designed shRNA oligonucleotides containing vector adapters, shRNA hairpin, sequencing primer, and shRNA-specific barcode are incorporated into a lentiviral vectors and amplified before being applied to 293FT cells for viral production. (b) After viral library selection (Figure 1), shRNA-specific barcodes are recovered from selected cells through 3-step PCR amplification. The resulting barcodes are then indexed with custom indexes for multiplex high-throughput sequencing.

Supplementary Figure 3: Verification of normality assumption for linear model

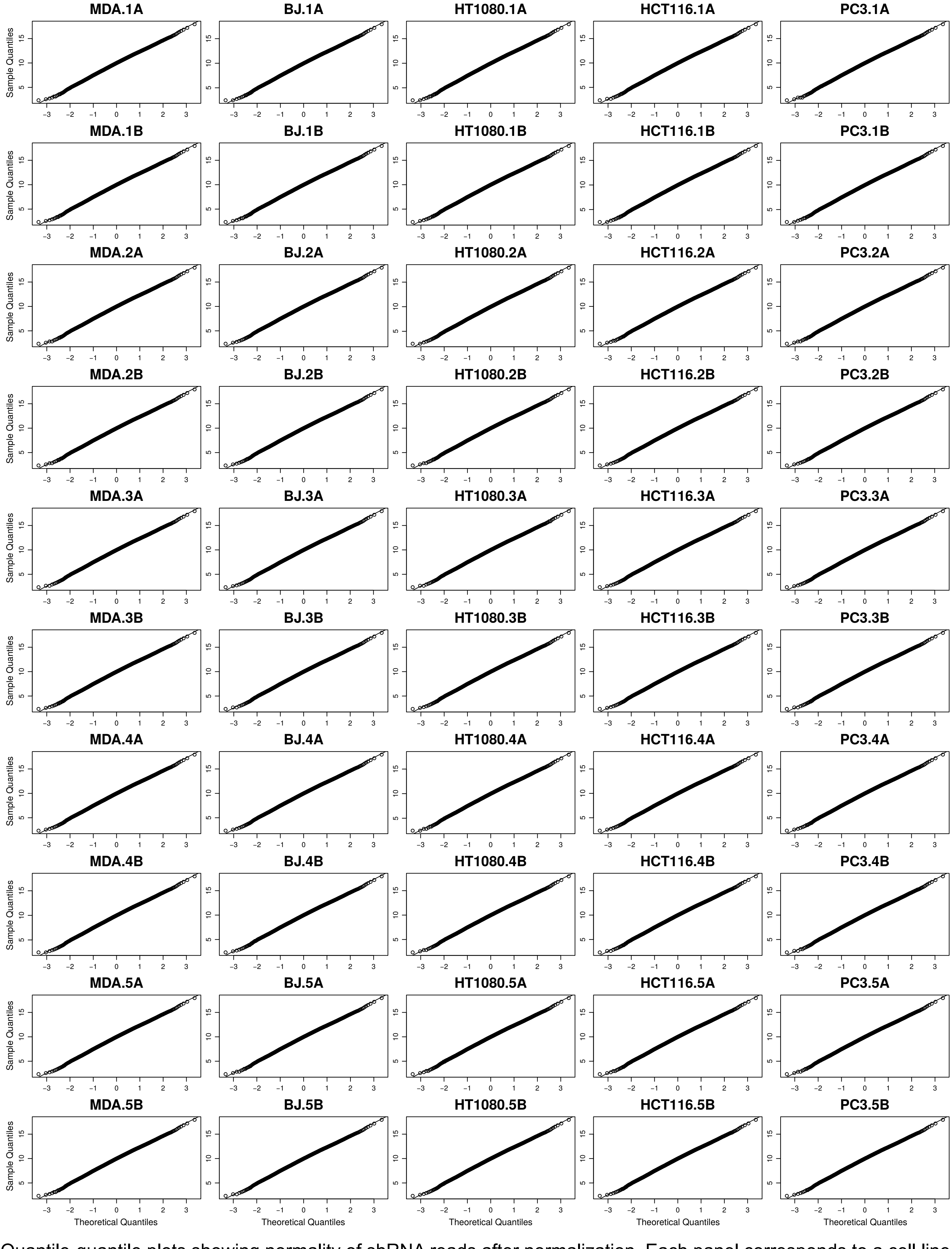

Quantile-quantile plots showing normality of shRNA reads after normalization. Each panel corresponds to a cell line, passage number (1-5), and biological replicate (A or B).

Supplementary Figure 4: Correlation of shRNA reads across replicates and passages

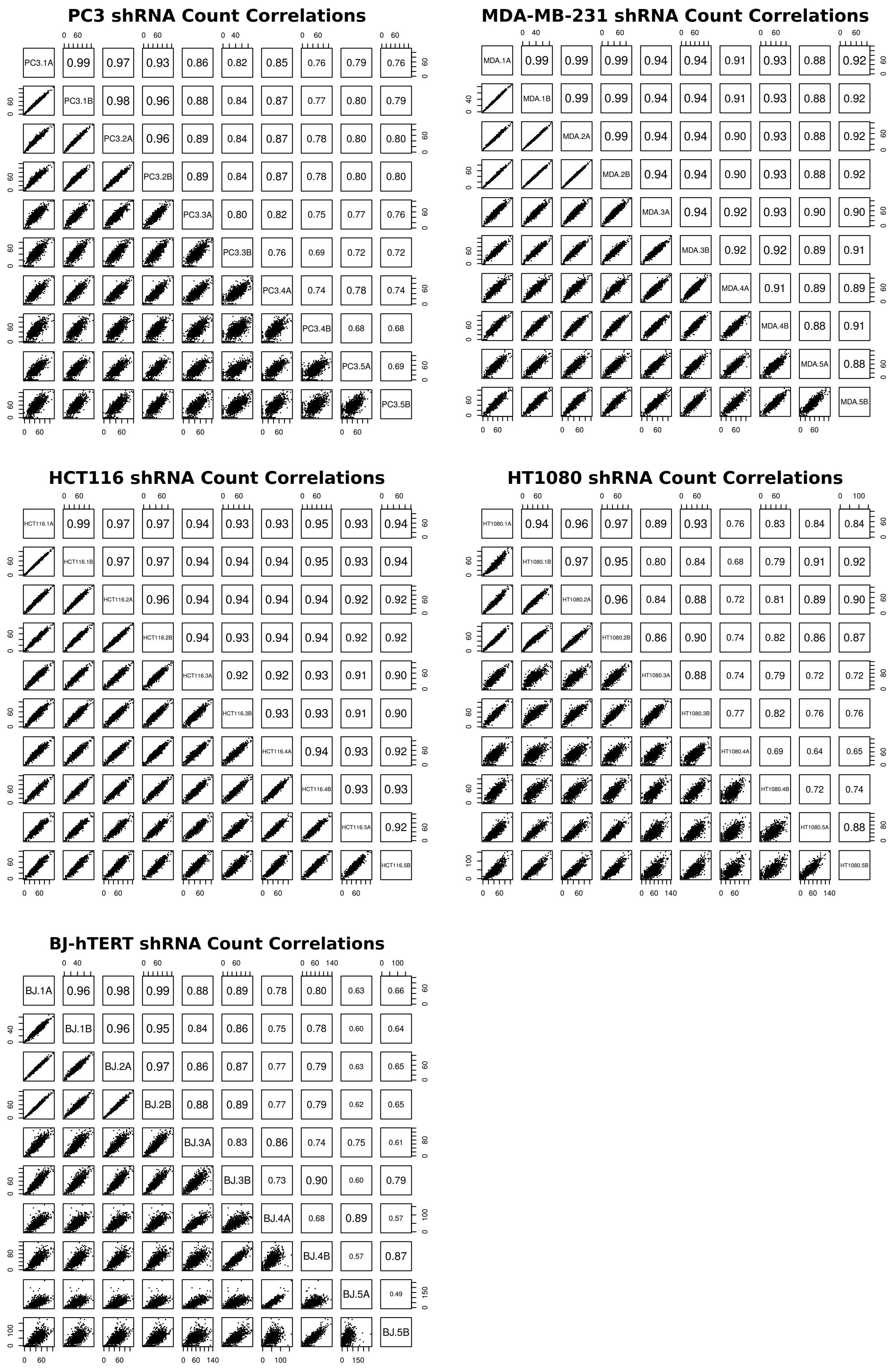

Each panel displays the correlation matrix across replicates and passages for each cell line. The lower triangle in each panel shows the scatter plot for shRNA reads. The upper triangle contains the correlation coefficient for the corresponding passage and replicate.

Supplemental Figure 5: Magnification of Figure 4

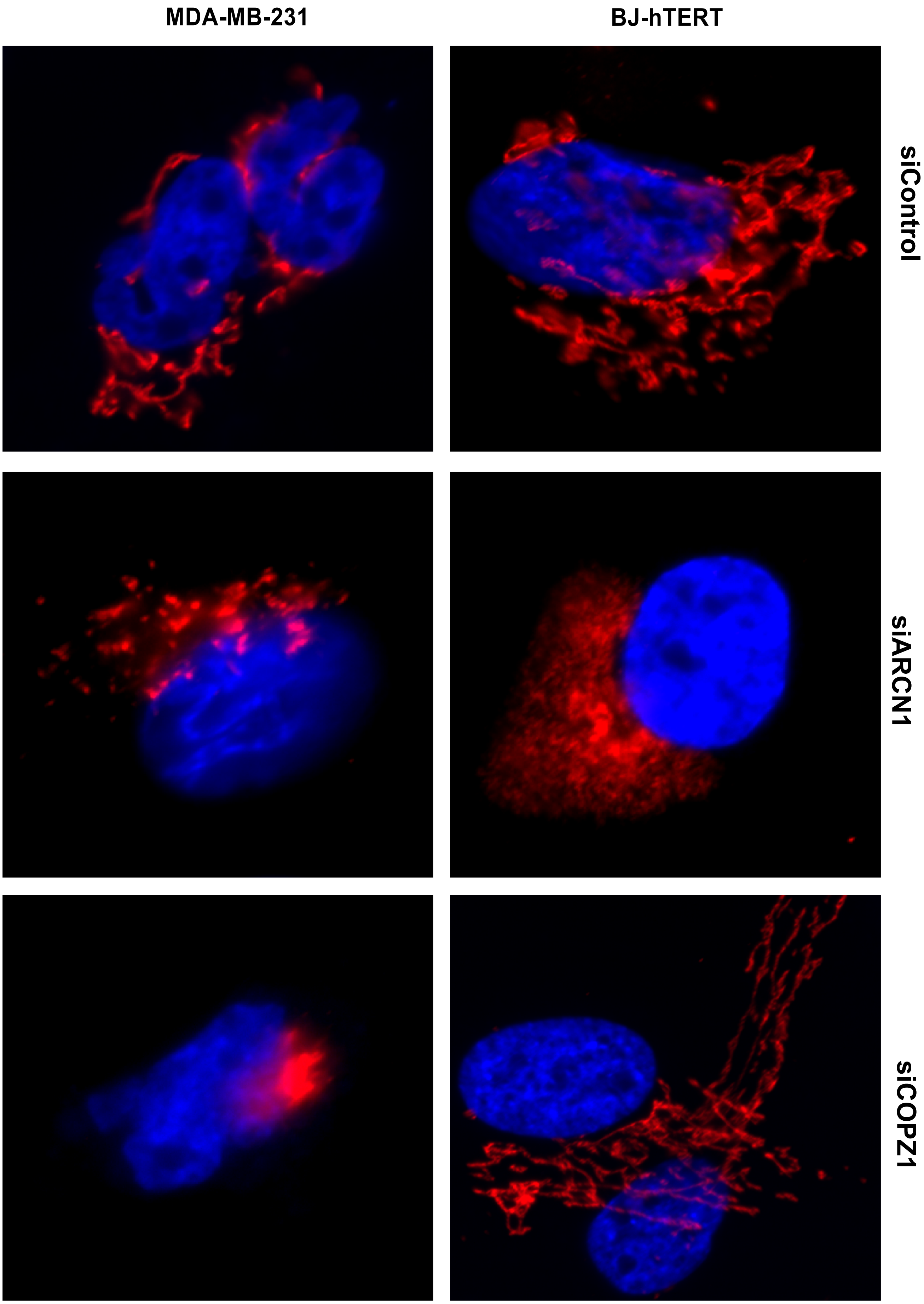

Disruption of Golgi structure after siRNA depletion of ARCN1 and COPZ1. Tubular structure in siControl sample indicates normal Golgi structure while ARCN1 produces dispersed vesicles in both normal and cancer cells. COPZ1 depletion produces abnormal Golgi structure in MDA-MB-231 but not in BJ-hTERT.

Supplementary Figure 6: Golgi disruption by ARCN1 depletion over time

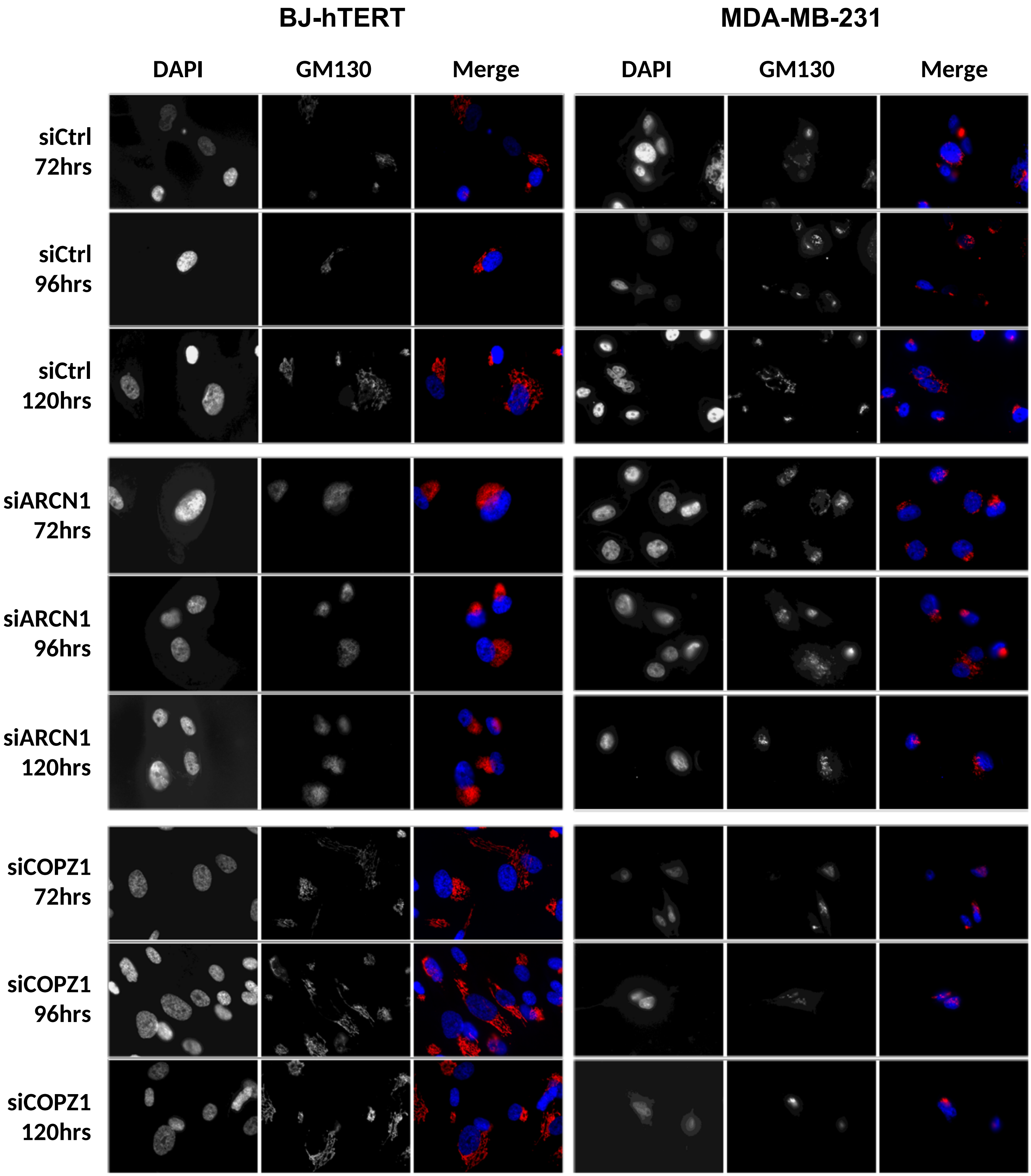

siRNA depletion with Control (top panel), ARCN1 (middle panel), or COPZ1 (bottom panel) siRNA in both BJ-hTERT (left panel) and MDA-MB-231 (right panel) monitored at 72, 96, and 120 hours post-transfection. Nuclei and Golgi were visualized with DAPI and anti-GM130 Abs respectively. Golgi disruption is seen as dispersion of GM130 staining while intact Golgi maintain tubule-like structure.

Supplimental Figure 7: RNA expression of RPL35A, DDX24, and ARCN1

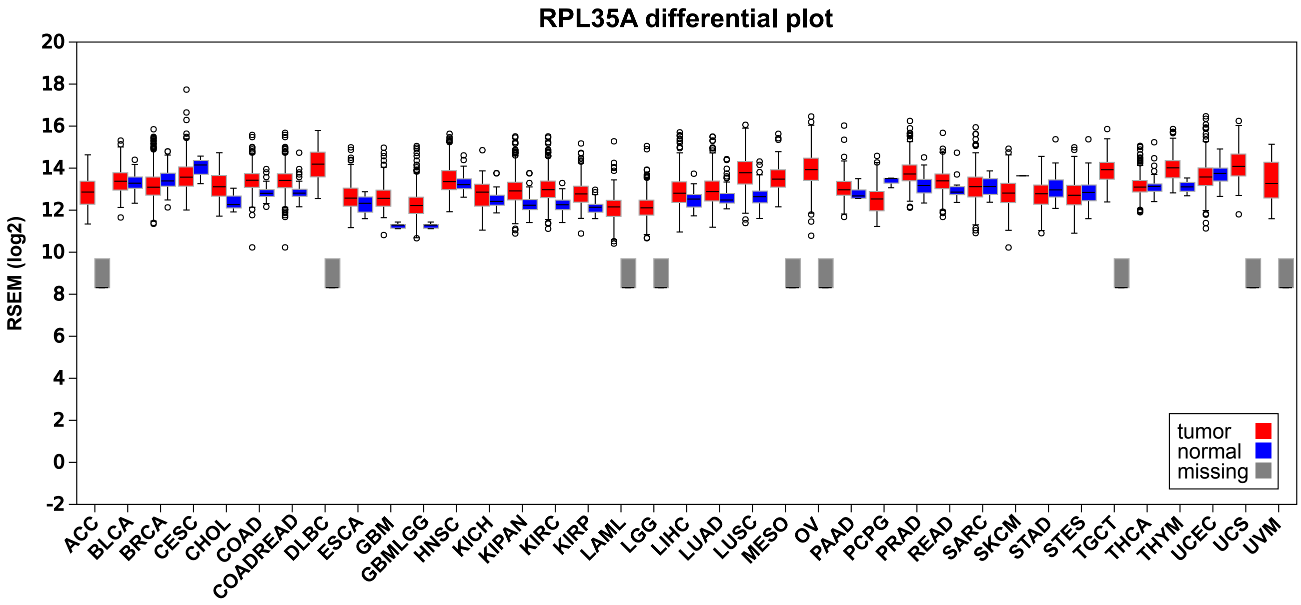

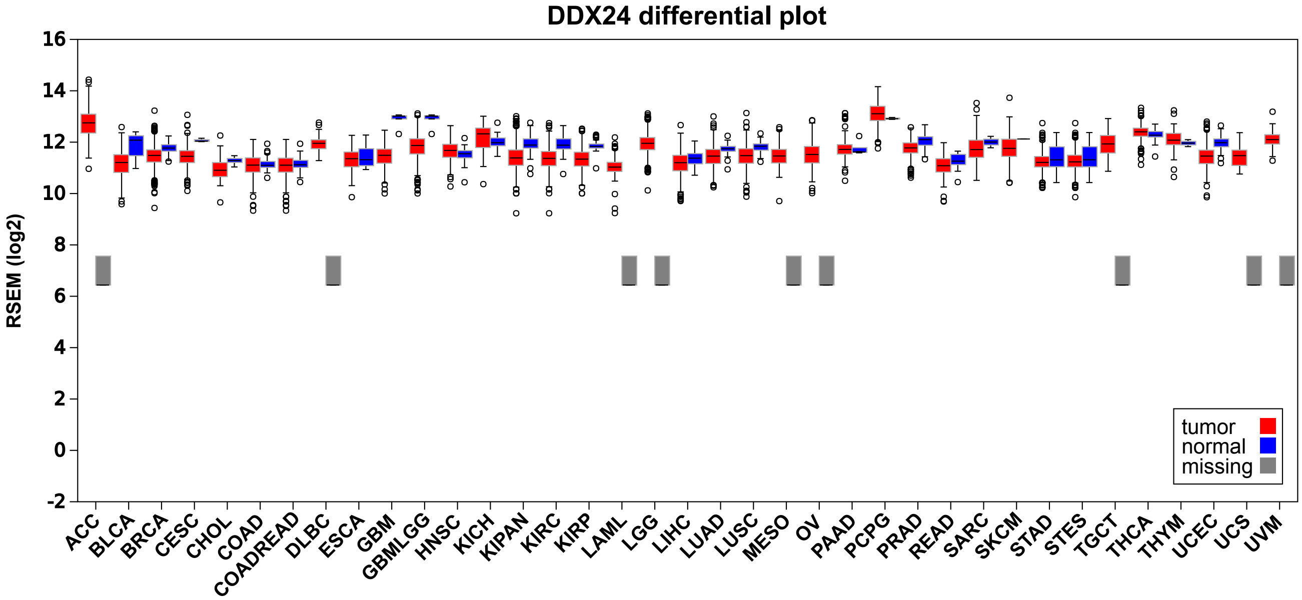

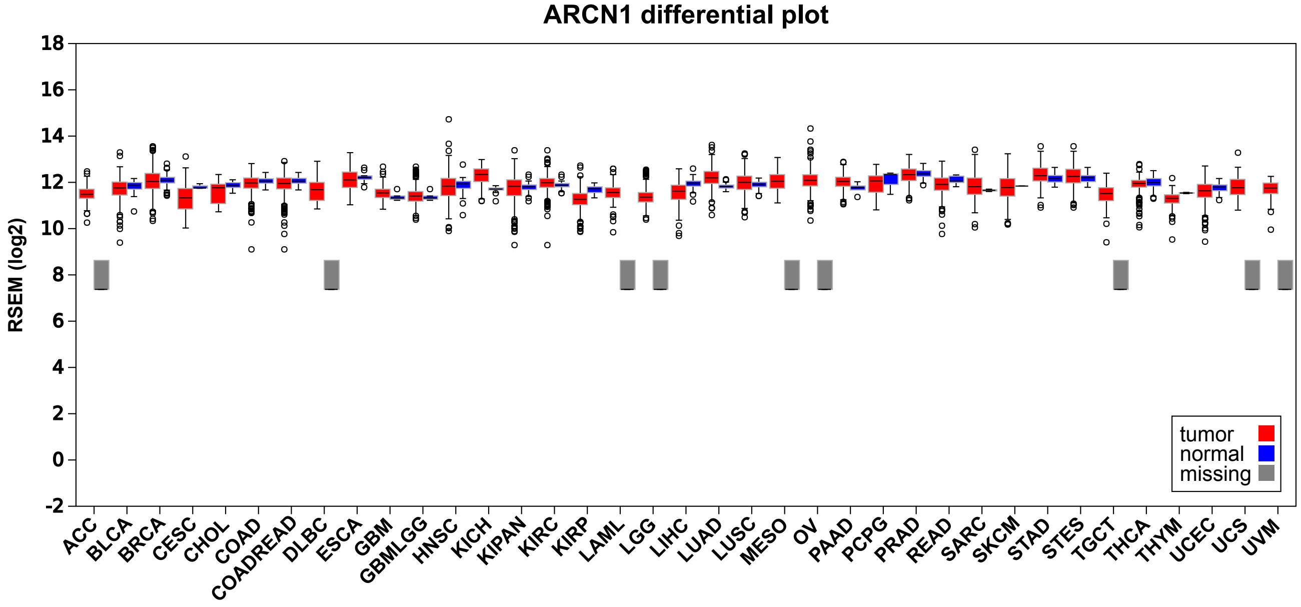

mRNA expression level of RPL35A, DDX24, and ARCN1. mRNA expression in tumor samples indicated by red box and whisker plots and corresponding notmal tissue expression level indicated in blue. Missing data indicated by gray bars. Description of samples in supplemental table 9. Data obtained from TCGA.

Description of cancer sample types for supplemental figure 7

ACC	Adrenocortical carcinoma
BLCA	Bladder Urothelial Carcinoma
BRCA	Breast invasive carcinoma
CESC	Cervical squamous cell carcinoma and endocervical adenocarcinoma
CHOL	Cholangiocarcinoma
COAD	Colon adenocarcinoma
COADREAD	Colorectal adenocarcinoma
DLBC	Lymphoid Neoplasm Diffuse Large B-cell Lymphoma
ESCA	Esophageal carcinoma
FPPP	FFPE Pilot Phase II
GBM	Glioblastoma multiforme
GBMLGG	Glioma
HNSC	Head and Neck squamous cell carcinoma
KICH	Kidney Chromophobe
KIPAN	Pan-kidney cohort (KICH+KIRC+KIRP)
KIRC	Kidney renal clear cell carcinoma
KIRP	Kidney renal papillary cell carcinoma
LAML	Acute Myeloid Leukemia
LGG	Brain Lower Grade Glioma
LIHC	Liver hepatocellular carcinoma
LUAD	Lung adenocarcinoma
LUSC	Lung squamous cell carcinoma
MESO	Mesothelioma
OV	Ovarian serous cystadenocarcinoma
PAAD	Pancreatic adenocarcinoma
PCPG	Pheochromocytoma and Paraganglioma
PRAD	Prostate adenocarcinoma
READ	Rectum adenocarcinoma
SARC	Sarcoma
SKCM	Skin Cutaneous Melanoma
STAD	Stomach adenocarcinoma
STES	Stomach and Esophageal carcinoma
TGCT	Testicular Germ Cell Tumors
THCA	Thyroid carcinoma
THYM	Thymoma
UCEC	Uterine Corpus Endometrial Carcinoma
UCS	Uterine Carcinosarcoma
UVM	Uveal Melanoma

Supplemental Figure 8

Figure 4B, Upper panel

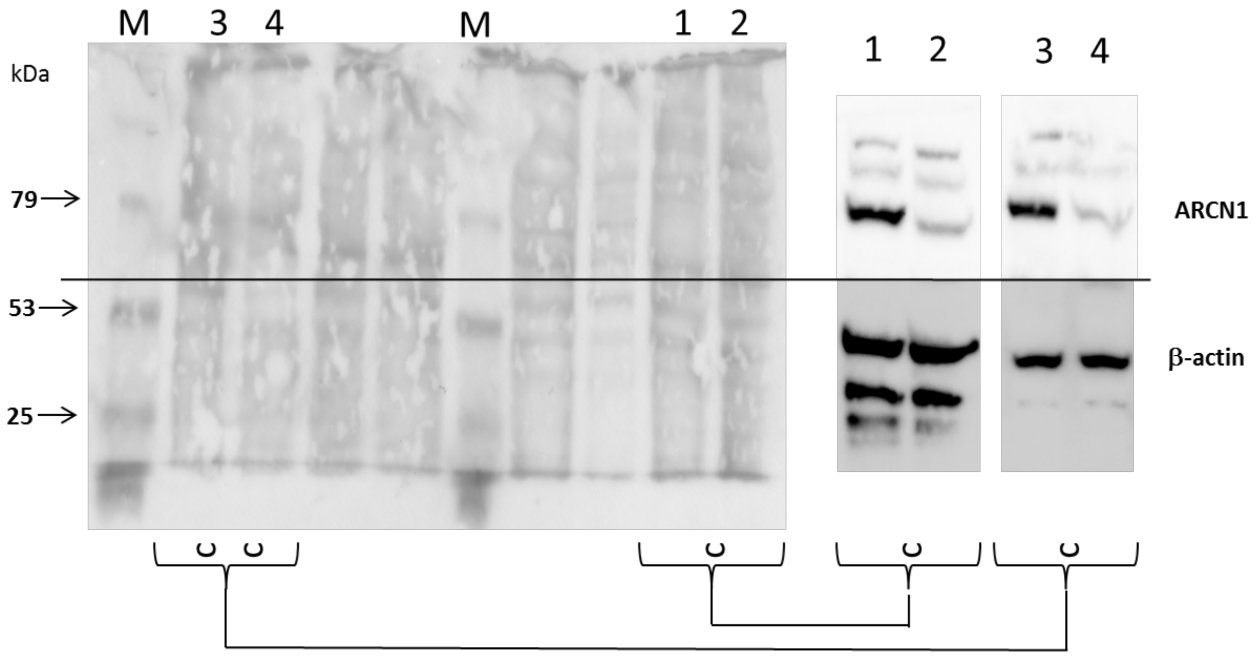

M –Precision Plus Protein™ All Blue Standards #1610373, BioRad
Gel: 8% Self Made Laemmli SDS Gel, Tris-Glycine

Figure 4B, Lower panel

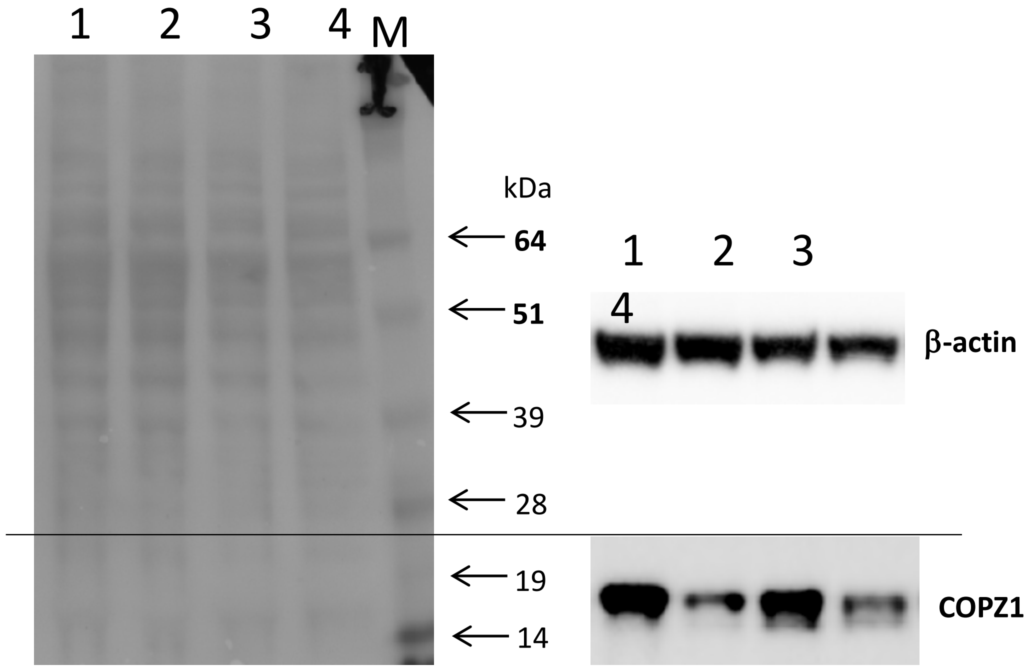

M – SeeBluePlus2 #LC5925, Thermo Scientific
Gel: 4 – 20% precast gradient gel, MOPS, Express Plus, #M42015, Gene Script

Supplemental Figure 8

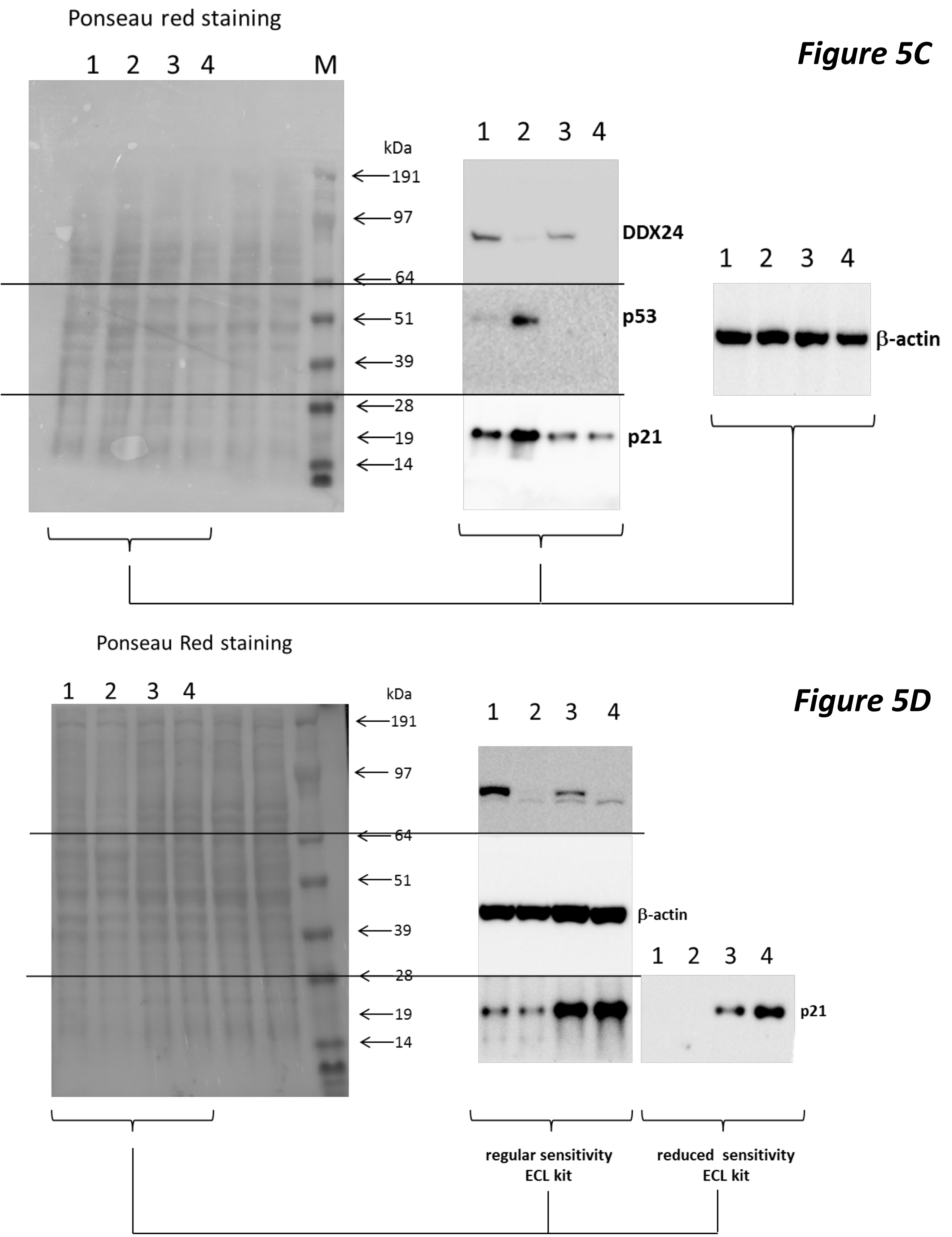

M – SeeBluePlus2 #LC5925, Thermo Scientific

Gel: 4 – 20% precast gradient gel, MOPS, Express Plus, #M42015, Gene Script

Supplemental Figure 8

Figure 6A

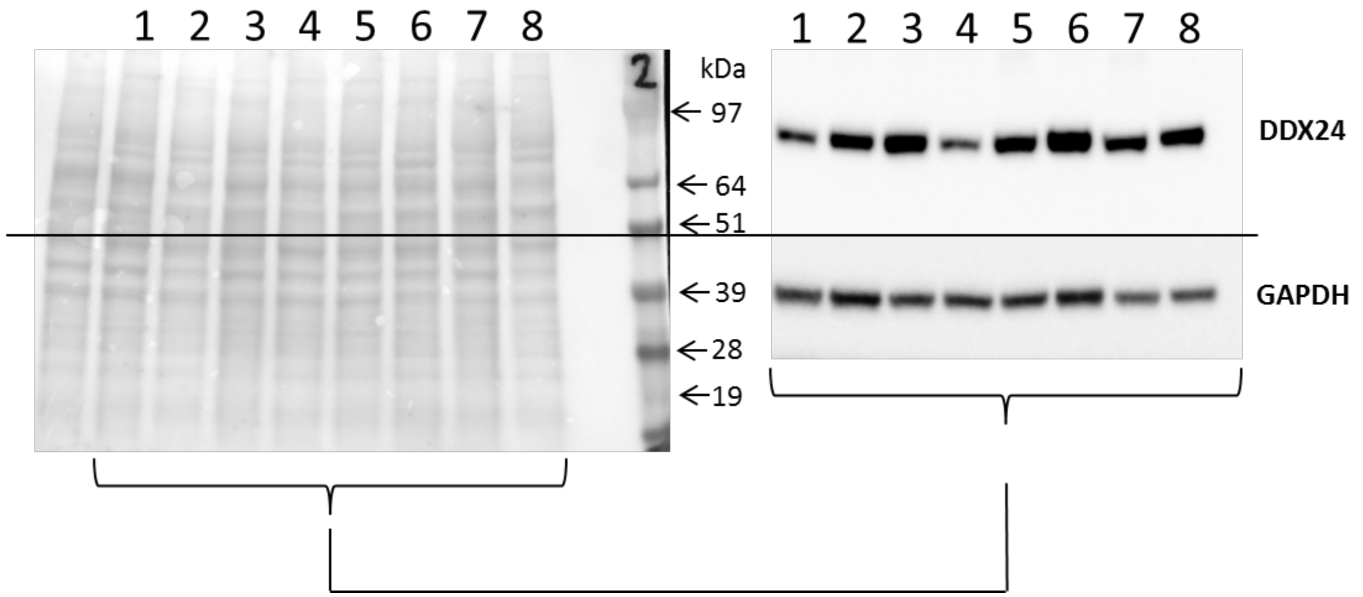

Figure 6B

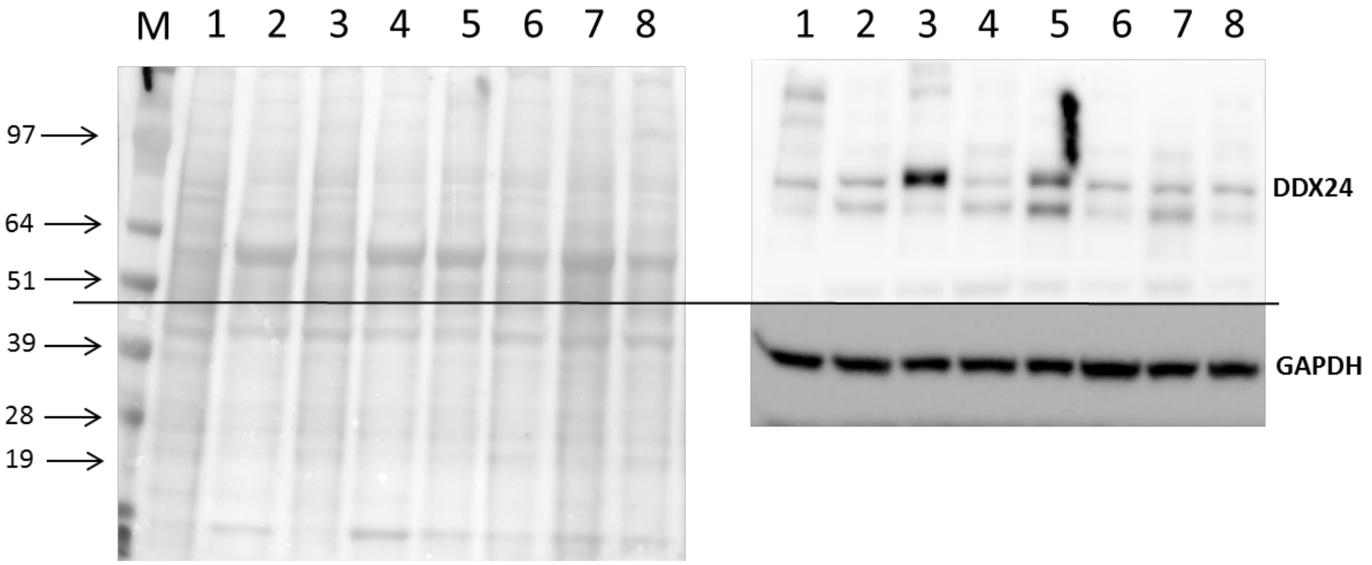

M – SeeBluePlus2 #LC5925, Thermo Scientific

Gel: 4 – 20% precast gradient gel, MOPS, Express Plus, #M42015, Gene Script

Supplemental Tables:

Supplemental Table S1: GO analysis results for hits from GSE screen

Supplemental Table S2: GO analysis identified 18 genes involved in RNA-splicing from list of GSE hits

Supplemental Table S3: GO analysis identified 21 genes involved in protein translation from GSE hits

Supplemental Table S4: Kegg Pathway search identified 8 genes from 221 GSE hits associated with PI3K-AKT signaling

Supplementary Table S5: Genes Identified with level of evidence indicated by color legend

Supplementary Table S6: Literature references for genes associated with cancer progression

Supplementary Table S7 List of primers used for amplification of the library

Supplementary Table S8: List of individual indexes

Supplemental Table S9: Results of siRNA screening with pooled siRNAs (GE Healthcare). Data represented relative to transfection with scrambled siRNA

Supplemental DataSets:

Supplemental DataSet S1. Genes targeted by GSEs enriched in BrdU-selected subsets in 2 or more tumor cell lines.

Supplementary DataSet S2: Sequences for 1274 shRNAs targeting 216 genes from GSE screen for which unique shRNAs designed.

Supplemental Table 1: GO analysis results for hits from GSE screen						
	Reference	upload_1				
Mapped IDs:	20814	179				
Unmapped IDs:	0	4				
Export results						
Displaying only results with P<0.05; click here to display all results						
	Homo sap	upload_1				
GO biological process complete	#	#	expected	Fold Enric	+/-	P value
translational initiation	232	14	2	> 5	+	1.50E-04
RNA splicing	338	18	2.91	> 5	+	9.16E-06
macromolecular complex disassemb	229	11	1.97	> 5	+	4.53E-02
translation	397	19	3.41	> 5	+	1.75E-05
peptide biosynthetic process	420	19	3.61	> 5	+	4.30E-05
mRNA processing	396	17	3.41	4.99	+	5.99E-04
amide biosynthetic process	480	20	4.13	4.84	+	6.52E-05
mRNA metabolic process	564	23	4.85	4.74	+	6.83E-06
peptide metabolic process	517	20	4.45	4.5	+	2.19E-04
RNA processing	705	26	6.06	4.29	+	4.09E-06
cellular component disassembly	493	17	4.24	4.01	+	1.21E-02
cellular amide metabolic process	666	22	5.73	3.84	+	6.65E-04
organonitrogen compound biosynth	1007	28	8.66	3.23	+	3.70E-04
intracellular protein transport	727	20	6.25	3.2	+	4.24E-02
single-organism intracellular transpo	1168	30	10.04	2.99	+	6.50E-04
intracellular transport	1353	32	11.64	2.75	+	1.45E-03
cellular protein localization	1138	26	9.79	2.66	+	4.42E-02
cellular macromolecule localization	1146	26	9.86	2.64	+	5.00E-02
establishment of localization in cell	1812	39	15.58	2.5	+	6.13E-04
protein localization	1689	35	14.53	2.41	+	7.89E-03
cellular localization	2174	45	18.7	2.41	+	1.48E-04
gene expression	3825	77	32.89	2.34	+	1.98E-10
macromolecule localization	2013	40	17.31	2.31	+	3.27E-03
cellular response to stress	1718	34	14.77	2.3	+	3.18E-02
macromolecular complex subunit or	2074	39	17.84	2.19	+	1.84E-02
cellular nitrogen compound metabol	5112	91	43.96	2.07	+	2.75E-10
RNA metabolic process	3373	60	29.01	2.07	+	8.25E-05
cellular nitrogen compound biosynth	3407	60	29.3	2.05	+	1.20E-04
organelle organization	2912	51	25.04	2.04	+	2.75E-03
nitrogen compound metabolic proce	5475	95	47.08	2.02	+	2.15E-10
cellular macromolecule biosynthetic	3576	62	30.75	2.02	+	1.10E-04
nucleic acid metabolic process	3874	66	33.32	1.98	+	5.78E-05
macromolecule biosynthetic process	3651	62	31.4	1.97	+	2.44E-04
cellular component organization	5066	85	43.57	1.95	+	1.22E-07
cellular component organization or b	5188	87	44.62	1.95	+	5.70E-08
nucleobase-containing compound m	4372	72	37.6	1.91	+	3.34E-05
heterocycle metabolic process	4573	75	39.33	1.91	+	1.49E-05
cellular aromatic compound metabo	4575	74	39.34	1.88	+	3.90E-05

cellular biosynthetic process	4493	72	38.64	1.86	+	1.11E-04
organic cyclic compound metabolic p	4818	76	41.43	1.83	+	6.49E-05
organic substance biosynthetic proce	4587	72	39.45	1.83	+	2.69E-04
biosynthetic process	4673	72	40.19	1.79	+	5.91E-04
cellular macromolecule metabolic pr	6753	103	58.08	1.77	+	3.58E-08
macromolecule metabolic process	7438	111	63.97	1.74	+	6.46E-09
localization	4683	67	40.27	1.66	+	3.58E-02
organic substance metabolic process	9008	124	77.47	1.6	+	1.53E-08
cellular metabolic process	8573	118	73.73	1.6	+	1.71E-07
primary metabolic process	8697	118	74.79	1.58	+	5.07E-07
metabolic process	9928	132	85.38	1.55	+	9.47E-09
cellular process	14147	156	121.66	1.28	+	1.90E-05
biological_process	16542	170	142.26	1.19	+	2.91E-05
Unclassified	4272	9	36.74	0.24	-	0.00E+00

	Supplemental Table 2: GO analysis identified 18 genes involved in RNA-splicing from list of GSE hits				
	**Gene ID**	**Mapped IDs**	**Gene Name**	**PANTHER Family/Subfamily**	**PANTHER Protein Class**
1	HUMAN|HGNC=8014|UniProtKB=P67809	YBX1	Nuclease-sensitive element-binding protein 1	NUCLEASE-SENSITIVE ELEMENT-BINDING PROTEIN 1 (PTHR11544:SF68)	-
2	HUMAN|HGNC=17066|UniProtKB=Q9UKV3	ACIN1	Apoptotic chromatin condensation inducer in the nucleus	APOPTOTIC CHROMATIN CONDENSATION INDUCER IN THE NUCLEUS (PTHR10799:SF637)	DNA helicase
3	HUMAN|HGNC=11161|UniProtKB=P62304	SNRPE	Small nuclear ribonucleoprotein E	SMALL NUCLEAR RIBONUCLEOPROTEIN E (PTHR11193:SF2)	mRNA splicing factor
4	HUMAN|HGNC=23136|UniProtKB=Q9BWG6	SCNM1	Sodium channel modifier 1	SODIUM CHANNEL MODIFIER 1 (PTHR32297:SF1)	-
5	HUMAN|HGNC=9188|UniProtKB=P30876	POLR2B	DNA-directed RNA polymerase II subunit RPB2	DNA-DIRECTED RNA POLYMERASE II SUBUNIT RPB2 (PTHR20856:SF7)	nucleotidyltransferase
6	HUMAN|HGNC=11155|UniProtKB=P08579	SNRPB2	U2 small nuclear ribonucleoprotein B''	U2 SMALL NUCLEAR RIBONUCLEOPROTEIN B'' (PTHR10501:SF21)	mRNA splicing factor
7	HUMAN|HGNC=23169|UniProtKB=Q9Y224	C14orf166	UPF0568 protein C14orf166	UPF0568 PROTEIN C14ORF166 (PTHR15924:SF9)	-
8	HUMAN|HGNC=14245|UniProtKB=O95149	SNUPN	Snurportin-1	SNURPORTIN-1 (PTHR13403:SF6)	-
9	HUMAN|HGNC=10768|UniProtKB=O75533	SF3B1	Splicing factor 3B subunit 1	SPLICING FACTOR 3B SUBUNIT 1 (PTHR12097:SF0)	mRNA splicing factor
10	HUMAN|HGNC=16939|UniProtKB=O95391	SLU7	Pre-mRNA-splicing factor SLU7	PRE-MRNA-SPLICING FACTOR SLU7 (PTHR12942:SF2)	mRNA splicing factor
11	HUMAN|HGNC=2746|UniProtKB=P17844	DDX5	Probable ATP-dependent RNA helicase DDX5	ATP-DEPENDENT RNA HELICASE DDX5-RELATED (PTHR24031:SF219)	RNA helicase
12	HUMAN|HGNC=16463|UniProtKB=O75400	PRPF40A	Pre-mRNA-processing factor 40 homolog A	PRE-MRNA-PROCESSING FACTOR 40 HOMOLOG A (PTHR11864:SF20)	-

13	HUMAN|HGNC=24217|UniProtKB=Q86X95	CIR1	Corepressor interacting with RBPJ 1	COREPRESSOR INTERACTING WITH RBPJ 1 (PTHR13151:SF2)	-
14	HUMAN|HGNC=5041|UniProtKB=P31943	HNRNPH1	Heterogeneous nuclear ribonucleoprotein H	HETEROGENEOUS NUCLEAR RIBONUCLEOPROTEIN H (PTHR13976:SF34)	ribosomal protein
15	HUMAN|HGNC=13058|UniProtKB=O95218	ZRANB2	Zinc finger Ran-binding domain-containing protein 2	ZINC FINGER RAN-BINDING DOMAIN-CONTAINING PROTEIN 2 (PTHR12999:SF7)	-
16	HUMAN|HGNC=17162|UniProtKB=Q9Y4Y9	LSM5	U6 snRNA-associated Sm-like protein LSm5	U6 SNRNA-ASSOCIATED SM-LIKE PROTEIN LSM5 (PTHR20971:SF0)	mRNA splicing factor
17	HUMAN|HGNC=23244|UniProtKB=P49756	RBM25	RNA-binding protein 25	RNA-BINDING PROTEIN 25 (PTHR18806:SF4)	RNA binding protein
18	HUMAN|HGNC=10530|UniProtKB=O00422	SAP18	Histone deacetylase complex subunit SAP18	HISTONE DEACETYLASE COMPLEX SUBUNIT SAP18 (PTHR13082:SF0)	chromatin/chromatin-binding protein

Supplemetal Table 3: GO analysis identified 21 genes involved in protein translation from GSE hits

Gene ID	Mapped IDs	Gene Name	PANTHER Family/Subfamily	PANTHER Protein Class
HUMAN|HGNC=17718|UniProtKB=P67812	SEC11A	Signal peptidase complex catalytic subunit SEC11A	SIGNAL PEPTIDASE COMPLEX CATALYTIC SUBUNIT SEC11A	-
HUMAN|HGNC=3285|UniProtKB=P23588	EIF4B	Eukaryotic translation initiation factor 4B	EUKARYOTIC TRANSLATION INITIATION FACTOR 4B (PTHR23236:SF2)	translation initiation factor
HUMAN|HGNC=10316|UniProtKB=P62829	RPL23	60S ribosomal protein L23	60S RIBOSOMAL PROTEIN L23 (PTHR11761:SF8)	ribosomal protein
HUMAN|HGNC=11325|UniProtKB=Q9UNL2	SSR3	Translocon-associated protein subunit gamma	TRANSLOCON-ASSOCIATED PROTEIN SUBUNIT GAMMA (PTHR13399:SF2)	-
HUMAN|HGNC=14480|UniProtKB=Q9NWU5	MRPL22	39S ribosomal protein L22, mitochondrial	39S RIBOSOMAL PROTEIN L22, MITOCHONDRIAL (PTHR13501:SF2)	ribosomal protein
HUMAN|HGNC=26121|UniProtKB=Q96EI5	TCEAL4	Transcription elongation factor A protein-like 4	TRANSCRIPTION ELONGATION FACTOR A PROTEIN-LIKE 4	transcription factor
HUMAN|HGNC=11330|UniProtKB=P30872	SSR1	Somatostatin receptor type 1	SOMATOSTATIN RECEPTOR TYPE 1 (PTHR24229:SF38)	G-protein coupled receptor
HUMAN|HGNC=3254|UniProtKB=Q9BY44	EIF2A	Eukaryotic translation initiation factor 2A	EUKARYOTIC TRANSLATION INITIATION FACTOR 2A (PTHR13227:SF0)	translation initiation factor
HUMAN|HGNC=23357|UniProtKB=Q9ULC4	MCTS1	Malignant T-cell-amplified sequence 1	MALIGNANT T-CELL-AMPLIFIED SEQUENCE 1 (PTHR22798:SF0)	receptor
HUMAN|HGNC=14049|UniProtKB=O60783	MRPS14	28S ribosomal protein S14, mitochondrial	28S RIBOSOMAL PROTEIN S14, MITOCHONDRIAL (PTHR19836:SF19)	ribosomal protein
HUMAN|HGNC=11332|UniProtKB=P32745	SSR3	Somatostatin receptor type 3	SOMATOSTATIN RECEPTOR TYPE 3 (PTHR24229:SF42)	G-protein coupled receptor
HUMAN|HGNC=10345|UniProtKB=P18077	RPL35A	60S ribosomal protein L35a	60S RIBOSOMAL PROTEIN L35A (PTHR10902:SF2)	ribosomal protein
HUMAN|HGNC=2240|UniProtKB=Q92905	COPS5	COP9 signalosome complex subunit 5	COP9 SIGNALOSOME COMPLEX SUBUNIT 5 (PTHR10410:SF6)	transcription factor
HUMAN|HGNC=11323|UniProtKB=P43307	SSR1	Translocon-associated protein subunit alpha	TRANSLOCON-ASSOCIATED PROTEIN SUBUNIT ALPHA (PTHR12924:SF1)	membrane traffic protein

HUMAN|HGNC=26274|UniProtKB=Q96I59	NARS2	Probable asparagine--tRNA ligase, mitochondrial	ASPARAGINE--TRNA LIGASE, MITOCHONDRIAL-RELATED (PTHR22594:SF34)	RNA binding protein
HUMAN|HGNC=10301|UniProtKB=P62913	RPL11	60S ribosomal protein L11	60S RIBOSOMAL PROTEIN L11 (PTHR11994:SF12)	ribosomal protein
HUMAN|HGNC=16945|UniProtKB=Q9H074	PAIP1	Polyadenylate-binding protein-interacting protein 1	POLYADENYLATE-BINDING PROTEIN-INTERACTING PROTEIN 1	RNA binding protein
HUMAN|HGNC=14513|UniProtKB=Q9Y2Q9	MRPS28	28S ribosomal protein S28, mitochondrial	28S RIBOSOMAL PROTEIN S28, MITOCHONDRIAL (PTHR13447:SF2)	-
HUMAN|HGNC=14512|UniProtKB=Q92552	MRPS27	28S ribosomal protein S27, mitochondrial	28S RIBOSOMAL PROTEIN S27, MITOCHONDRIAL (PTHR21393:SF0)	ribosomal protein
HUMAN|HGNC=16635|UniProtKB=P82673	MRPS35	28S ribosomal protein S35, mitochondrial	28S RIBOSOMAL PROTEIN S35, MITOCHONDRIAL (PTHR13490:SF0)	ribosomal protein
HUMAN|HGNC=14493|UniProtKB=Q9Y6G3	MRPL42	39S ribosomal protein L42, mitochondrial	39S RIBOSOMAL PROTEIN L42, MITOCHONDRIAL (PTHR13450:SF1)	ribosomal protein

Supplmentary Table 4: Kegg Pathway search identified 8 genes from 221 GSE hits associated with PI3K-AKT signaling		
Gene Symbol	Gene Info	KEGG Pathway Title
EIF4B	eukaryotic translation initiation factor 4B	PI3K-Akt signaling pathway; RNA transport; mTOR signaling pathway; Proteoglycans in cancer
RHEB	Ras homolog enriched in brain	PI3K-Akt signaling pathway; mTOR signaling pathway; Thyroid hormone signaling pathway; AMPK signaling pathway; Insulin signaling pathway; Choline metabolism in cancer
GRB2	growth factor receptor-bound protein 2	PI3K-Akt signaling pathway; MAPK signaling pathway; Osteoclast differentiation; Choline metabolism in cancer; Chemokine signaling pathway; Dorso-ventral axis formation; Alcoholism; Pathways in cancer; Chronic myeloid leukemia; Focal adhesion; Fc epsilon RI signaling pathway; Insulin signaling pathway; Hepatitis C; Acute myeloid leukemia; Ras signaling pathway; Gap junction; T cell receptor signaling pathway; B cell receptor signaling pathway; Proteoglycans in cancer; Renal cell carcinoma; Non-small cell lung cancer; Jak-STAT signaling pathway; GnRH signaling pathway; Glioma; Prostate cancer; Signaling pathways regulating pluripotency of stem cells; Prolactin signaling pathway; Viral carcinogenesis; Endometrial cancer; ErbB signaling pathway; FoxO signaling pathway; Natural killer cell mediated cytotoxicity; Neurotrophin signaling pathway; Estrogen signaling pathway; Hepatitis B; MicroRNAs in cancer
IL2RG	interleukin 2 receptor, gamma	PI3K-Akt signaling pathway; Cytokine-cytokine receptor interaction; Endocytosis; Jak-STAT signaling pathway; Measles; HTLV-I infection; Inflammatory bowel disease (IBD); Primary immunodeficiency
HSP90AB1	heat shock protein 90kDa alpha (cytosolic), class B member 1	PI3K-Akt signaling pathway; Protein processing in endoplasmic reticulum; NOD-like receptor signaling pathway; Progesterone-mediated oocyte maturation; Prostate cancer; Antigen processing and presentation; Pathways in cancer; Estrogen signaling pathway

SPP1	secreted phosphoprotein 1	PI3K-Akt signaling pathway; Toll-like receptor signaling pathway; Focal adhesion; ECM-receptor interaction
DCN	decorin	PI3K-Akt signaling pathway; Proteoglycans in cancer; TGF-beta signaling pathway
CD44	CD44 molecule (Indian blood group)	PI3K-Akt signaling pathway; ECM-receptor interaction; Hematopoietic cell lineage; Epstein-Barr virus infection; Proteoglycans in cancer; MicroRNAs in cancer; Shigellosis

Supplementary Table 5: Genes Identified with level of evidence indicated by color legend

Color Legend

Greater than 0 in all 4 cell lines

Greater than 0 in 3 of 4 cell lines

Greater than 0 in 2 of 4 cell lines

Greater than 0.1 in 1 of 4 cell lines

Gene_ID	Gene_Name	HCT116-Lrstat	HCT116-Slope	HT1080-Lrstat	HT1080-Slope	MDA-Lrstat	MDA-Slope	PC3-Lrstat	PC3-Slope	BJ-Slope
ARCN1	Coatomer subunit delta	0.12861	-81.542	0.03	-46.192	0.080138	-68.6417	0.00137	-18.1417	-10.2583
HMGB1	High mobility group protein B1	0.00194	-63.017	0.0105	-76.825	0.004052	-66.75	0.906342	-244.908	54.73333
LOC644100	-	0.02246	-112.82	0.0243	-118.23	8.25E-05	-67.5167	0.001175	-76.1583	-64.525
RABIF	Guanine nucleotide exchange factor MSS4	0.2235	-143.05	0.4111	-205.42	0.128671	-111.825	0.00897	-43.975	15.4
SARNP	SAP domain-containing ribonucleoprotein	0.01252	-63.458	0.0093	-58.958	0.044519	-101.917	0.3096	-231.533	19.25833
SENP7	Sentrin-specific protease 7	0.15227	-62.733	0.0308	-33.45	0.609844	-124.05	3.370883	-301.692	-0.79167
AGFG1	Arf-GAP domain and FG repeat-containing protein 1	0	41.9917	0.0569	-68.042	0.002876	-31.175	0.02382	-50.5	20.075
ATP6V1E1	V-type proton ATPase subunit E 1	0	-94.475	0.0033	-128.09	0.048077	-170.95	0.059616	-179.742	-111.725
CORO1C	Coronin-1C	0	-21.358	0.0428	-110.15	0.052586	-113.55	0.016183	-85.5083	46.21667
HNRNPH1	Heterogeneous nuclear ribonucleoprotein H	0	-18.25	0.1195	-169.58	0.051106	-126.225	0.398095	-245.708	60.725
IL2RG	Cytokine receptor common subunit gamma	0.00508	-48.35	0	15.275	1.235997	-277.542	0.022652	-67.5417	-31.9167

KLHDC8A	Kelch domain-containing protein 8A	0	12.0917	0.0618	-57.433	0.308127	-115.467	0.611858	-166.4	-5.9
PRPF40A	Pre-mRNA-processing factor 40 homolog A	0.02839	-112.96	0.0199	-108.85	0	78.8	0.052754	-128.308	73.66667
SETD5	SET domain-containing protein 5	0	-0.9083	0.0997	-121.23	0.048413	-98.1917	0.015317	-80.7083	56.46667
UTP11L	U3 small nucleolar RNA-associated protein 11	0	19.3	0.0536	-67.25	0.006658	-30.075	0.070815	-80.1167	-8.33333
AGBL1	Cytosolic carboxypeptidase 4	0	8.38333	0.1953	-128.97	0	75.64167	0.655649	-207.708	34.66667
COPZ1	Coatomer subunit zeta-1	0.00058	-51.392	0	16.4333	0.024606	-82.2667	0	-36.95	45.69167
DDX24	ATP-dependent RNA helicase DDX24	0	-2.425	0	452.775	0.020173	-63.4083	0.131735	-160.733	-7.79167
EIF4B	Eukaryotic translation initiation factor 4B	0	-20.433	0.2248	-224.97	0.030773	-140.725	0	3.875	96.79167
GRB2	Growth factor receptor-bound protein 2	0	-32.317	0.0077	-59.525	0.231014	-167.85	0	-24.7	-34.8917
KCTD1	BTB/POZ domain-containing protein KCTD1	0.09771	-62.683	0	47.85	0	-2.18333	0.046891	-47.8167	-8.43333
LOC100505894	-	0.01508	-41.767	0	31.7083	0	4.866667	0.011925	-41.5083	-24.6167
LOC727919	-	0	63.875	0	228.158	0.000131	-94.275	0.06875	-166.933	-91.1917
NIT2	Omega-amidase NIT2	0	91.9167	0.2658	-123.62	0	111.25	0.001222	-9.425	-1.325
OCIAD2	OCIA domain-containing protein 2	0	-75.775	0	-88.308	0.016999	-163.383	0.110477	-220.183	-129.2
OXSR1	Serine/threonine-protein kinase OSR1	0	-21.417	0	180.025	0.000434	-121.7	0.02293	-169.583	114.3667

PDIA3	Protein disulfide-isomerase A3	0	16.9167	0	15.875	0.3885	-141.592	0.126365	-106.042	-44.5167
PLEKHA7	Pleckstrin homology domain-containing family A member 7	0	-23.642	0.1224	-119.34	0.007987	-67.5833	0	-16.95	-49.0833
RPL35A	60S ribosomal protein L35a	0.08467	-117.84	0	45.9583	0.316173	-161.517	0	-36.8917	-65.2083
RPL36AP7	-	0.10976	-81.342	0	232.117	0	31.18333	9.70E-05	-7.31667	-4.96667
SLC25A35	Solute carrier family 25 member 35	0	250.942	0	397	0.031912	-63.5417	0.504695	-208.875	18.675
SORBS2	Sorbin and SH3 domain-containing protein 2	0	-13.192	0.0244	-69.05	0.000502	-27.1833	0	6.666667	-20.1417
TERF2IP	Telomeric repeat-binding factor 2-interacting protein 1	0.00021	-10.708	0	36.8083	0	62.7	0.018244	-46.2917	6.566667
XPO5	Exportin-5	0	-5.1	0.0204	-42.908	0.109217	-67.9333	0	24.50833	-10.8833
ATRX	Transcriptional regulator ATRX	0	66.2	0.1794	-79.358	0	153.8167	0	91.38333	25.775
CDC26P1	-	0	-9.3583	0	40.425	0	3.275	0.112387	-125.758	-34.1583
LSM5	U6 snRNA-associated Sm-like protein LSm5	0	0.3	0.1261	-48.167	0	46.06667	0	14.85	3.925
MAP3K4	Mitogen-activated protein kinase kinase kinase 4	0	6.525	0	33.6083	0	74.03333	0.359337	-96.3083	-16.6917
METTL18	Histidine protein methyltransferase 1 homolog	0	-11.867	0	246.325	0.176803	-99.0083	0	-6.7	24.48333
PCBD2	Pterin-4-alpha-carbinolamine dehydratase 2	0	51.575	0.3111	-154.42	0	160.1083	0	15.81667	-21.1167
PSMC1P1	-	0	31.3833	0	193.658	0	-36.5083	0.165339	-288.35	-190.542
PUM1	Pumilio homolog 1	0	61.5583	0	-21.158	0	5.875	0.144704	-194.758	-128.917

RHEB	GTP-binding protein Rheb	0	57.5917	0	-55.858	0	-10.1333	0.153889	-181.8	73.89167
SPP1	Osteopontin	0	-52.383	0.2257	-123.83	0	22.85833	0	-35.2167	-59.4

Supplementary Table 6: Literature references for genes associated with cancer progression

Gene_ID	Gene_Name	Reference
ARCN1	Coatomer subunit delta	Kim, H. S. et al. Systematic identification of molecular subtype-selective vulnerabilities in non-small-cell lung cancer. Cell 155, 552-566 (2013).
HMGB1	High mobility group protein B1	Wang, X. et al. The Role of HMGB1 Signaling Pathway in the Development and Progression of Hepatocellular Carcinoma: A Review. Int J Mol Sci 16, 22527-22540 (2015).
ARL14EPL	ADP ribosylation factor like GTPase 14 effector protein like	
RABIF	Guanine nucleotide exchange factor MSS4	
SARNP	SAP domain-containing ribonucleoprotein	Hashii, Y. et al. A novel partner gene CIP29 containing a SAP domain with MLL identified in infantile myelomonocytic leukemia. Leukemia 18, 1546-1548 (2004).
SENP7	Sentrin-specific protease 7	Bawa-Khalfe, T. et al. Differential expression of SUMO-specific protease 7 variants regulates epithelial-mesenchymal transition. Proc. Natl. Acad. Sci. U.S.A. 109, 17466-17471 (2012).
AGFG1	Arf-GAP domain and FG repeat-containing	
ATP6V1E1	V-type proton ATPase subunit E 1	Sreekumar, B. K. et al. Polarization of the vacuolar adenosine triphosphatase delineates a transition to high-grade pancreatic intraepithelial neoplasm lesions. Pancreas 43, 1256-1263 (2014).
CORO1C	Coronin-1C	Wu, L. et al. [Increased coronin-1C expression is related to hepatocellular carcinoma invasion and metastasis]. Zhonghua Gan Zang Bing Za Zhi 18, 516-519 (2010).
HNRNPH1	Heterogeneous nuclear ribonucleoprotein H	Lefave, C. V. et al. Splicing factor hnRNPH drives an oncogenic splicing switch in gliomas. EMBO J. 30, 4084-4097 (2011).
IL2RG	Cytokine receptor common subunit gamma	Agarwal, A. et al. Functional RNAi screen targeting cytokine and growth factor receptors reveals oncorequisite role for interleukin-2 gamma receptor in JAK3-mutation-positive leukemia. Oncogene 34, 2991-2999 (2015).
KLHDC8A	Kelch domain-containing protein 8A	Mukasa, A. et al. Mutant EGFR is required for maintenance of glioma growth in vivo, and its ablation leads to escape from receptor dependence. Proc. Natl. Acad. Sci. U.S.A. 107, 2616-2621 (2010).
PRPF40A	Pre-mRNA-processing factor 40 homolog A	
SETD5	SET domain-containing protein 5	
UTP11	U3 small nucleolar RNA-associated protein	
AGBL1	Cytosolic carboxypeptidase 4	
COPZ1	Coatomer subunit zeta-1	Shtutman, M. et al. Tumor-specific silencing of COPZ2 gene encoding coatomer protein complex subunit ζ 2 renders tumor cells dependent on its paralogous gene COPZ1. Proc. Natl. Acad. Sci. U.S.A. 108, 12449-12454 (2011).
DDX24	ATP-dependent RNA helicase DDX24	

EIF4B	Eukaryotic translation initiation factor 4B	Degen, M., Barron, P., Natarajan, E., Widlund, H. R. & Rheinwald, J. G. RSK activation of translation factor eIF4B drives abnormal increases of laminin γ2 and MYC protein during neoplastic progression to squamous cell carcinoma. PLoS ONE 8, e78979 (2013).
GRB2	Growth factor receptor-bound protein 2	Ahmed, Z. et al. Grb2 monomer-dimer equilibrium determines normal versus oncogenic function. Nat Commun 6, 7354 (2015).
KCTD1	BTB/POZ domain-containing protein KCTD1	Li, X. et al. KCTD1 suppresses canonical Wnt signaling pathway by enhancing β-catenin degradation. PLoS ONE 9, e94343 (2014).
TMEM161B-AS1	TMEM161B antisense RNA 1	
LOC727919	NADH:ubiquinone oxidoreductase complex assembly factor 2 pseudogene	
NIT2	Omega-amidase NIT2	Zheng, B., Chai, R. & Yu, X. Downregulation of NIT2 inhibits colon cancer cell proliferation and induces cell cycle arrest through the caspase-3 and PARP pathways. Int. J. Mol. Med. 35, 1317-1322 (2015).
OCIAD2	OCIA domain-containing protein 2	Nagata, C. et al. Increased expression of OCIA domain containing 2 during stepwise progression of ovarian mucinous tumor. Pathol. Int. 62, 471-476 (2012).
OXSR1	Serine/threonine-protein kinase OSR1	
PDIA3	Protein disulfide-isomerase A3	Liao, C. J. et al. Glucose-regulated protein 58 modulates β-catenin protein stability in a cervical adenocarcinoma cell line. BMC Cancer 14, 555 (2014).
PLEKHA7	Pleckstrin homology domain-containing family A member 7	Tille, J. C. et al. The Expression of the Zonula Adhaerens Protein PLEKHA7 Is Strongly Decreased in High Grade Ductal and Lobular Breast Carcinomas. PLoS ONE 10, e0135442 (2015).
RPL35A	60S ribosomal protein L35a	Kroes, R. A. et al. The identification of novel therapeutic targets for the treatment of malignant brain tumors. Cancer Lett. 156, 191-198 (2000).
RPL36AP7	ribosomal protein L36a pseudogene 7	
SLC25A35	Solute carrier family 25 member 35	
SORBS2	Sorbin and SH3 domain-containing protein 2	Backsch, C. et al. An integrative functional genomic and gene expression approach revealed SORBS2 as a putative tumour suppressor gene involved in cervical carcinogenesis. Carcinogenesis 32, 1100-1106 (2011).
TERF2IP	Telomeric repeat-binding factor 2-interacting protein 1	Potrony, M. et al. Update in genetic susceptibility in melanoma. Ann Transl Med 3, 210 (2015).
XPO5	Exportin-5	Li, Y., Wang, X., He, B., Cai, H. & Gao, Y. Downregulation and tumor-suppressive role of XPO5 in hepatocellular carcinoma. Mol. Cell. Biochem. 415, 197-205 (2016).

ATRX	Transcriptional regulator ATRX	Lee, J. C. et al. Alternative lengthening of telomeres and loss of ATRX are frequent events in pleomorphic and dedifferentiated liposarcomas. Mod. Pathol. 28, 1064-1073 (2015).
CDC26P1	cell division cycle 26 pseudogene 1	
LSM5	U6 snRNA-associated Sm-like protein LSm5	
MAP3K4	Mitogen-activated protein kinase kinase kinase 4	Whitmarsh, A. J. & Davis, R. J. Role of mitogen-activated protein kinase kinase 4 in cancer. Oncogene 26, 3172-3184 (2007).
METTL18	Histidine protein methyltransferase 1	
PCBD2	Pterin-4-alpha-carbinolamine dehydratase	
PSMC1P1	proteasome 26S subunit, ATPase 1	
PUM1	Pumilio homolog 1	Fernandez, S. et al. miR-340 inhibits tumor cell proliferation and induces apoptosis by targeting multiple negative regulators of p27 in non-small cell lung cancer. Oncogene 34, 3240-3250 (2015).
RHEB	GTP-binding protein Rheb	Wang, Y. et al. Inhibition of MAPK pathway is essential for suppressing Rheb-Y35N driven tumor growth. Oncogene, (2016).
SPP1	Osteopontin	Lin, J. et al. Osteopontin (OPN/SPP1) isoforms collectively enhance tumor cell invasion and dissemination in esophageal adenocarcinoma. Oncotarget 6, 22239-22257 (2015).

Supplemental Table 7: List of primers used for amplification of the library

Primer Name	Primer Sequences
FwdHTS	TTCTCTGGCAAGCAAAAGACGGCATA
RevHTS	TGCCATTTGTCTCGAGGTCGAGAA
FwdGex	CAAGCAGAAGACGGCATACGAGA
Rev_Ind_1	ACACGACGCTCTTCCGATCT**ACAAGCTA**CGAAACCCCAAACGCACGAA
Seq2N_AD	AATGATACGGCGACCACCGAGATCTACACTCTTTCCCTACACGACGCTCTTCCGATCT
Red letters:	Primer Rev_Ind_1 represents seriens of index primes. All 50 indexes are in Supp Table 10. The 8 base index sequence marked with red.

Supplementary Table 8: List of individual indexes

Index Num.	Sample Name	Index seq
1	MDA-1A	ACAAGCTA
2	MDA-1B	AAACATCG
3	MDA-2A	ACATTGGC
4	MDA-2B	ACCACTGT
5	MDA-3A	AACGTGAT
6	MDA-3B	CGCTGATC
7	MDA-4A	CAGATCTG
8	MDA-4B	ATGCCTAA
9	MDA-5A	CTGTAGCC
10	MDA-5B	AGTACAAG
11	MDA-6A	CATCAAGT
14	BJ-1A	AACCGAGA
15	BJ-1B	AACGCTTA
16	BJ-2A	AAGACGGA
17	BJ-2B	AAGGTACA
18	BJ-3A	ACACAGAA
19	BJ-3B	ACAGCAGA
20	BJ-4A	ACCTCCAA
21	BJ-4B	ACGCTCGA
22	BJ-5A	ACGTATCA
23	BJ-5B	ACTATGCA
26	HT1080-1A	AGCAGGAA
27	HT1080-1B	AGTCACTA
28	HT1080-2A	ATCCTGTA
29	HT1080-2B	ATTGAGGA
30	HT1080-3A	CAACCACA
31	HT1080-3B	CAAGACTA
32	HT1080-4A	CAATGGAA
33	HT1080-4B	CACTTCGA
34	HT1080-5A	CAGCGTTA
38	HCT116-1A	CCGTGAGA
39	HCT116-1B	CCTCCTGA
40	HCT116-2A	CGAACTTA
41	HCT116-2B	CGACTGGA
42	HCT116-3A	CGCATACA
43	HCT116-3B	CTCAATGA
44	HCT116-4A	CTGAGCCA
45	HCT116-4B	CTGGCATA
46	HCT116-5A	GAATCTGA
47	HCT116-5B	GACTAGTA
51	PC3-1A	GCGAGTAA
52	PC3-1B	GCTAACGA
53	PC3-2A	GCTCGGTA
54	PC3-2B	GGAGAACA

55	PC3-3A	GGTGCGAA
56	PC3-3B	GTACGCAA
57	PC3-4A	GTCGTAGA
58	PC3-4B	GTCTGTCA
59	PC3-5A	GTGTTCTA
60	PC3-5B	TAGGATGA

Supplemetal Table 9 siRNA screenings results

siRNA	BJ-hTERT	HCT-116	MB-231	PC-3
Mock	1	1	1	1
Non-targeting	0.993637	1.076347	1.145371	1.236729
Empty	0.995182	0.940182	1.05333	1.053895
AGBL1	0.993353	0.829061	0.94132	0.968732
HNRNPH1	1.008916	0.706796	0.887928	0.950942
PCBD2	0.994845	0.973945	0.957894	1.045638
SETD5	1.000087	0.748977	0.898195	1.077305
AGFG1	0.994808	0.938085	1.028644	0.9287
IL2RG	0.996655	0.963529	1.293999	1.290599
PDIA3	0.992133	0.928903	1.11852	1.121697
SLC25A35	0.995125	0.996245	1.01256	1.077171
ARCN1	0.989305	0.265602	0.323694	0.297051
KCTD1	0.997719	0.992288	1.070425	1.118217
PLEKHA7	0.995435	1.110114	0.985993	1.312232
SORBS2	0.997753	1.056556	1.089335	1.01474
ATP6V1E1	0.993027	1.116636	0.982125	0.955134
KLHDC8A	1.005215	0.895708	1.265156	1.104444
PRPF40A	0.9932	1.085609	0.980048	0.980877
SPP1	0.997114	1.164841	1.005836	1.182003
ATRX	0.991238	0.935701	1.260971	0.848173
LSM5	0.99214	0.942326	0.982554	0.571907
PUM1	0.992634	0.990456	1.012888	0.99408
TERF2IP	1.000324	1.102796	1.102099	1.008148
CORO1C	0.987569	1.175916	1.003253	0.879636
MAP3K4	0.996622	1.006132	1.108993	0.934333
RABIF	0.998564	1.01153	1.184535	1.032891
UTP11L	0.998573	0.81696	0.843181	0.863122
DDX24	0.993122	0.652864	0.593598	0.567028
METTL18	1.009398	1.076623	1.0784	1.05731
RHEB	0.996713	1.104943	1.284004	0.906698
XPO5	0.987849	1.166598	1.029362	0.925374
EIF4B	0.995085	0.917745	0.95623	0.87972
NIT2	0.996144	1.103389	1.151813	1.239495
RPL35A	0.8349	0.255282	0.29914	0.343375
GRB2	0.991738	0.581017	1.017937	0.979098
OCIAD2	0.993775	1.002994	1.212914	1.177656
SARNP	0.994299	0.978375	1.19879	0.995627
HMGB1	0.987477	0.892343	1.056406	0.952099
OXSR1	1.003865	0.969629	1.03246	1.096614
SENP7	0.995039	1.018165	0.988002	1.114387
